# Internal sensory neurons regulate stage-specific growth in *Drosophila*

**DOI:** 10.1242/dev.200440

**Published:** 2022-10-28

**Authors:** Yuya Ohhara, Naoki Yamanaka

**Affiliations:** ^1^ School of Food and Nutritional Sciences, University of Shizuoka, Shizuoka 422-8526, Japan; ^2^ Graduate School of Integrated Pharmaceutical and Nutritional Sciences, University of Shizuoka, Shizuoka 422-8526, Japan; ^3^ Department of Entomology, Institute for Integrative Genome Biology, University of California, Riverside, Riverside, CA 92521, USA

**Keywords:** Gustatory receptors, Insulin-like peptides, Multidendritic neurons, *Drosophila*

## Abstract

Animals control their developmental schedule in accordance with internal states and external environments. In *Drosophila* larvae, it is well established that nutrient status is sensed by different internal organs, which in turn regulate production of insulin-like peptides and thereby control growth. In contrast, the impact of the chemosensory system on larval development remains largely unclear. Here, we performed a genetic screen to identify gustatory receptor (Gr) neurons regulating growth and development, and found that *Gr28a*-expressing neurons are required for proper progression of larval growth. *Gr28a* is expressed in a subset of peripheral internal sensory neurons, which directly extend their axons to insulin-producing cells (IPCs) in the central nervous system. Silencing of *Gr28a*-expressing neurons blocked insulin-like peptide release from IPCs and suppressed larval growth during the mid-larval period. These results indicate that *Gr28a*-expressing neurons promote larval development by directly regulating growth-promoting endocrine signaling in a stage-specific manner.

## Teaser

**Highlighted Article:** A subset of gustatory receptor-expressing internal sensory neurons in *Drosophila* innervates insulin-producing cells and regulates larval development in a stage-specific manner.

## INTRODUCTION

During animal development, members of the insulin-like peptide (ILP) family, including insulin-like growth factors in vertebrates and ILPs in insects, play an essential role in nutrient-dependent growth (Garofalo, 2002; Hietakangas and Cohen, 2009; Okamoto and Yamanaka, 2015; Tennessen and Thummel, 2011). In the fruit fly *Drosophila melanogaster*, eight ILPs (ILP1-8) are encoded in the genome, among which ILP2, ILP3 and ILP5 are produced in neuroendocrine cells called insulin-producing cells (IPCs) located in the central nervous system (CNS) (Ikeya et al., 2002; Okamoto and Yamanaka, 2015). ILPs released from IPCs systemically promote insulin signaling and growth during larval development (Garofalo, 2002; Hietakangas and Cohen, 2009; Okamoto and Yamanaka, 2015; Tennessen and Thummel, 2011). ILP production in IPCs is dependent on nutrient status, and the most crucial nutrient cue for ILP production is protein/amino acids. Under protein/amino acid-rich conditions, the fat body, an insect adipose/liver-like tissue, secretes various insulinotropic peptides, including Stunted, Growth-blocking peptides and CCHamide-2, to remotely stimulate ILP production in IPCs (Delanoue et al., 2016; Koyama and Mirth, 2016; Meschi et al., 2019; Sano et al., 2015). By contrast, protein/amino acid starvation blocks insulinotropic peptide secretion and stimulates insulinostatic Eiger release from the fat body (Agrawal et al., 2016; Delanoue et al., 2016; Koyama and Mirth, 2016; Meschi et al., 2019; Sano et al., 2015). Amino acids are also sensed by glial cells, and the downstream cholinergic neurons promote *ILP5* transcription in IPCs (Okamoto and Nishimura, 2015). Moreover, IPCs themselves can sense leucine through the amino acid transporters Juvenile hormone Inducible-21 and Minidiscs to promote ILP2 secretion and growth (Manière et al., 2016; Ziegler et al., 2018). Along with the protein/amino acid-dependent signaling pathways, a fat body-derived leptin-like peptide called Unpaired 2 stimulates ILP release and body growth in response to high-fat and high-sugar diets (Rajan and Perrimon, 2012).

In addition to these internal nutrient sensing mechanisms, *Drosophila* larvae can sense available nutrients and other environmental cues through the chemosensory system. Gustatory receptor (Gr) family is the best-characterized taste and chemosensory receptor repertoire, which encodes seven-transmembrane receptors that function in detection of sugars, noxious chemicals, and many other nutritional and environmental cues (Chen and Dahanukar, 2020; Joseph and Carlson, 2015). *Drosophila* genome has 60 Gr genes that encode 68 proteins through alternative splicing (Robertson et al., 2003), and expression patterns of these Grs in the larval chemosensory organs have been characterized by using the Gal4/UAS system (Choi et al., 2016; Kwon et al., 2011). In the larval stage, Grs are expressed in the sensory neurons in dorsal, ventral and posterior pharyngeal sense organs (DPS, VPS and PPS, respectively) and external chemosensory organs termed terminal organ ganglion (TOG) and dorsal organ ganglion (DOG) (Choi et al., 2016; Kwon et al., 2011). The gustatory receptor neurons (GRNs) extend their axons to the subesophageal zone (SEZ), a part of the CNS in which gustatory and sensory inputs are first processed and further transferred to higher brain centers (Chen and Dahanukar, 2020; Hückesfeld et al., 2015). Furthermore, loss-of-function and electrophysiological studies have revealed the importance of Grs in gustatory perception. For example, Gr43a acts as a sugar receptor during the larval stage (Mishra et al., 2013), whereas sugar responses in the adult stage depend on several Grs such as Gr5a, Gr61a and Gr64a-f (Dahanukar et al., 2007; Fujii et al., 2015; Jiao et al., 2008; Wang et al., 2004). Noxious stimuli such as caffeine are also mediated by Grs, including Gr8a, Gr33a, Gr66a and Gr93a (Dweck and Carlson, 2020; Lee et al., 2009, 2012; Moon et al., 2006, 2009). Gr28 clade genes (*Gr28a* and five *Gr28b* splicing variants) are involved in perception and behavioral outputs of various environmental cues, including temperature, light, ribonucleotides and plant-derived chemicals (Mishra et al., 2018a,b; Ni et al., 2013; Sang et al., 2019; Xiang et al., 2010). *Gr28a* and *Gr28b* are expressed not only in external and pharyngeal sensory neurons, but also in trachea-associated peripheral neurons and stomatogastric neurons (Mishra et al., 2018a; Qian et al., 2018; Thorne and Amrein, 2008). Although such expression patterns indicate potential involvement of GRNs in internal nutrient sensing in *Drosophila* larvae, the impact of GRNs on larval development and ILP production remains unknown.

Here, we screened a collection of *Gr-Gal4* lines to identify GRNs that regulate larval development. We found that *Gr28a-Gal4*-positive neurons are required for proper progression of larval growth. Further genetic analyses revealed that *Gr28a-*expressing body wall neurons innervate IPCs to promote ILP release and thereby regulate larval growth during mid-larval development. These body wall neurons may thus serve as internal sensory neurons that regulate *Drosophila* larval development in a stage-specific manner.

## RESULTS

### *Gr28a-Gal4*-positive neurons regulate larval development

**Fig. 1.**
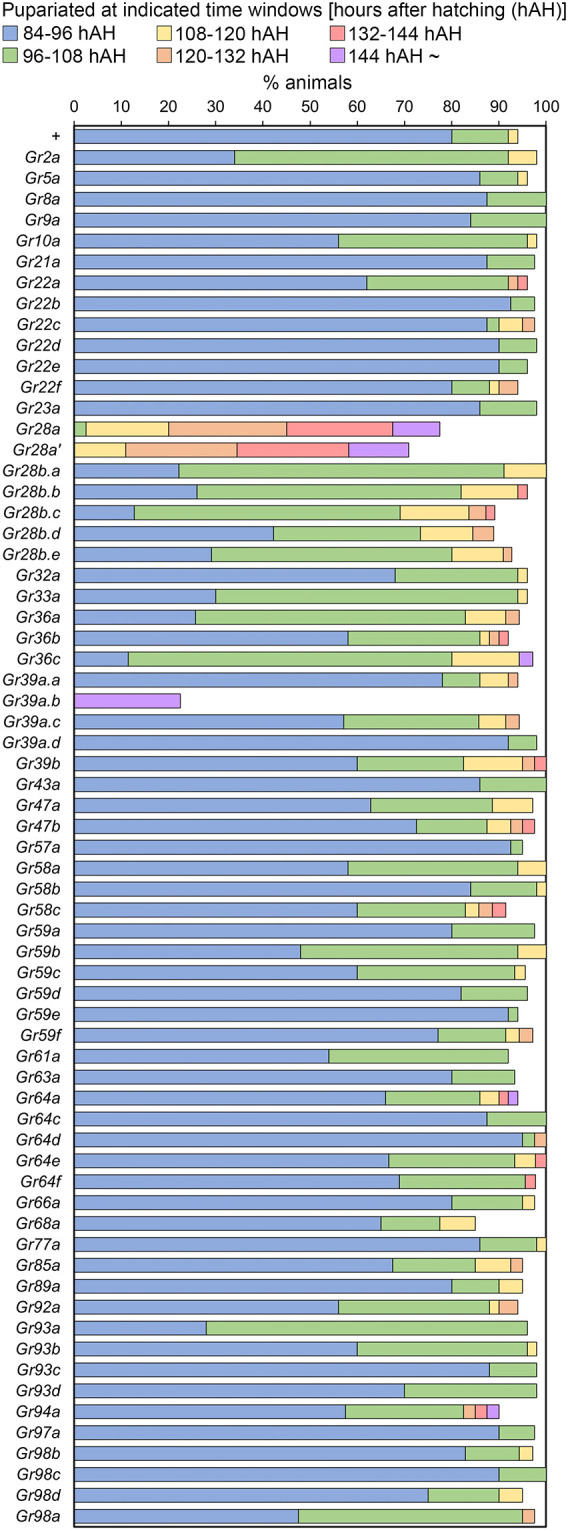
**GRN developmental timing screen results.** We screened 66 *Gr-Gal4* lines to identify GRNs that regulate larval development. *UAS-Kir2.1* was driven by *Gr-Gal4* to silence neuronal activities of *Gr-Gal4-*expressing neurons (*Gr>Kir2.1*). Percentages of animals of each genotype pupariated within indicated time windows are shown. Only Gr gene names are indicated on the left. + indicates the control possessing only *UAS-Kir2.1* (*+>Kir2.1*). Sample sizes are 35-65 for each genotype (see Table S1).

To identify Gr-expressing neurons regulating larval development, we performed a genetic screen using the Gal4/UAS system. We screened 66 *Gr-Gal4* lines with regulatory sequences derived from corresponding Gr genes (Table S1). Each *Gr-Gal4* line was crossed with *UAS-Kir2.1*, which expresses an inwardly rectifying potassium channel under the control of UAS (Johns et al., 1999), to silence neuronal activities of the Gr-expressing neurons in their progeny (*Gr>Kir2.1*). In control animals, which possess *UAS-Kir2.1* alone (*+>Kir2.1*), 80% pupariated between 84 h and 96 h after hatching (hAH), and 92% of them pupariated by 108 hAH (Fig. 1; Table S1). Compared with control, many *Gr-Gal4* lines induced varying degrees of developmental delay when crossed to *UAS-Kir2.1*, but two *Gr-Gal4* lines showed fundamental effects. First, *Gr28a>Kir2.1* animals showed a significant delay in pupariation, with more than half of them undergoing pupariation after 120 hAH, and more than 20% did not successfully pupariate (Fig. 1; Table S1). This phenotype was observed in two independent *Gr28a-Gal4* lines, which have distinct Gal4 insertion sites on the second and third chromosomes (Fig. 1; Table S1). These results indicate that *Gr28a*-expressing neurons are involved in the regulation of larval growth. In addition, more than 70% of *Gr39a.b>Kir2.1* animals showed lethality at the larval stage (Fig. 1; Table S1). Interestingly, however, *Gr39a.b-Gal4* is highly expressed in the proventriculus (Fig. S1), suggesting that this phenotype is potentially caused by impairment of the digestive system function. We therefore focused on *Gr28a*-expressing neurons hereafter and performed further genetic analyses to reveal their functions.

### *Gr28a-Gal4* is expressed in body wall-associated and stomatogastric neurons

**Fig. 2.**
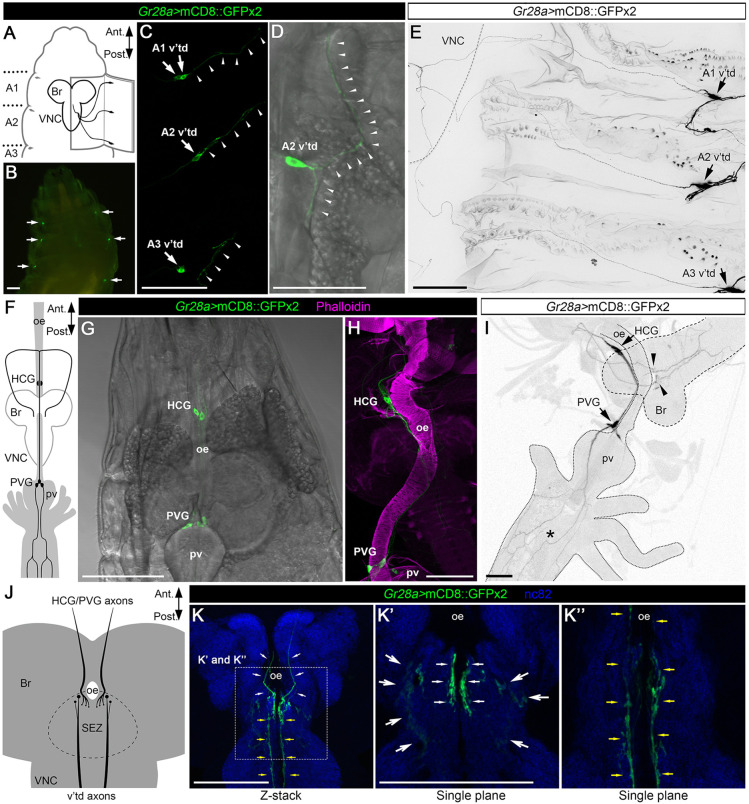
**Projection patterns of *Gr28a-Gal4*-positive v'td, HCG and PVG neurons.** (A) Schematic of the anterior larval body wall and v'td neurons. (B) *Gr28a-Gal4* expression in v'td neurons. *Gr28a-Gal4* expression was visualized by two copies of *UAS-mCD8::GFP*. The anterior larval body wall of a late second instar larva was captured using a fluorescent stereomicroscope. *Gr28a-Gal4*-positive v'td neurons are indicated by arrows. (C,D) Dendrites of *Gr28a*-expressing v'td neurons associated with tracheal tracts. Projections of confocal stacks of the body wall of late second instar larvae are shown. *Gr28a-Gal4* expression was visualized by two copies of *UAS-mCD8::GFP*. Cell bodies and dendrites of *Gr28a-Gal4*-positive v'td neurons are indicated by arrows and arrowheads, respectively. (E) Projection of confocal stacks of *Gr28a*-expressing v'td neurons projecting to the CNS of a late second instar larva. *Gr28a-Gal4* expression was visualized by two copies of *UAS-mCD8::GFP*. The CNS outline is indicated by a dashed line. Arrows indicate cell bodies of *Gr28a*-expressing v'td neurons. (F) Schematic of the CNS and digestive tract. (G,H) *Gr28a-Gal4* is expressed in HCG and PVG neurons on the foregut. A section (G) and a projection of confocal stacks (H) of *Gr28a-*expressing neurons in the HCG and PVG of late second instar larvae are shown. *Gr28a-Gal4* expression was visualized by two copies of *UAS-mCD8::GFP* (green). F-actin was stained by phalloidin (magenta in H). (I) *Gr28a*-expressing HCG and PVG neurons extend their dendrites and axons to the midgut and CNS, respectively. Projections of confocal stacks of *Gr28a-*expressing neurons in the HCG and PVG of late second instar larvae are shown. *Gr28a-Gal4* expression was visualized by two copies of *UAS-mCD8::GFP*. Cell bodies and axon termini of *Gr28a-*expressing neurons are indicated by arrows and arrowheads, respectively. The midgut with *Gr28a-Gal4*-positive dendrites is indicated by an asterisk. The outlines of the CNS and intestine are indicated by dashed and dotted lines, respectively. (J) Schematic of the larval brain. (K-K″) Axons of *Gr28a*-expressing neurons terminate on the SEZ. A projection (K) and sections (K′,K″) of confocal stacks of the brain of a late second instar larva are shown. *Gr28a-Gal4* expression was visualized by two copies of *UAS-mCD8::GFP* (green). A pre-synapse marker Bruchpilot was stained by nc82 antibody (blue). Yellow and white arrows indicate axon tracks of *Gr28a-Gal4*-positive v'td and HCG/PVG neurons, respectively. K′ and K″ show the magnified sections of the boxed area in K. Note that axon termini from the pharyngeal and external sensory neurons are also visible in the lateral SEZ (bigger arrows in K′). Ant, anterior; Br, brain; HCG, hypocerebral ganglion; oe, esophagus; Post, posterior; pv, proventriculus; PVG, proventricular ganglion; SEZ, subesophageal zone; VNC, ventral nerve cord. Scale bars: 100 µm (B,C,E,G-I,K-K″); 50 µm (D).

We next observed the expression pattern of *Gr28a-Gal4* by using *UAS-mCD8::GFPx2*, which expresses two copies of membrane-tethered GFP under the control of UAS. We confirmed that *Gr28a-Gal4* is expressed in multidendritic neurons on the body wall named v'td neurons (Fig. 2A-C) as previously reported (Mishra et al., 2018a; Qian et al., 2018; Thorne and Amrein, 2008). *Gr28a-Gal4*-positive v'td neurons are located on the ventrolateral body wall in abdominal segment 1 (A1), A2 and A3 (Fig. 2A-C). Two pairs of *Gr28a-Gal4*-positive v'td neurons, referred to as v'td1 and v'td2 neurons, were observed in the A1 segment, whereas a pair of *Gr28a-Gal4*-positive v'td2 neurons was detected in A2 and A3 (Fig. 2A-C) (Qian et al., 2018). Dendrites of *Gr28a-Gal4*-positive v'td neurons are associated with the tracheal branches (Fig. 2D), and their axons extend to the central brain through the ventral nerve cord (VNC) (Fig. 2E). Furthermore, we confirmed that *Gr28a-Gal4* was expressed in a subset of stomatogastric neurons on the esophagus and proventriculus, referred to as the hypocerebral ganglion (HCG) and proventricular ganglion (PVG), respectively (Fig. 2F-I) (Miroschnikow et al., 2018). *Gr28a-Gal4*-positive HCG and PVG neurons extend their dendrites on the midgut (Fig. 2I), and their axons project to the central brain through the esophagus tract (Fig. 2F,I). Both v'td and HCG/PVG neurons terminate on the SEZ, where these two clusters show distinct terminal loci (Fig. 2J-K″). *Gr28a-Gal4* is also expressed in the pharyngeal and external gustatory neurons and midgut cells (Fig. S2) (Mishra et al., 2018a). This *Gr28a-Gal4* expression pattern was observed in both 1st and 2nd instars (Fig. S2), suggesting that *Gr28a-Gal4* expression is relatively constant at least during the first two instars. Furthermore, when the *UAS-mCD8::GFPx2* reporter was driven by *Gr28a^GAL4^*, in which *Gr28a* was deleted and replaced with a Gal4-3xP3-RFP cassette (Fig. S3), GFP signals were detected in A1-A3 v'td, HCG and PVG neurons, and *Gr28a^GAL4^*-positive axon termini were detected in the SEZ (Fig. S3). These results collectively indicate that v'td and stomatogastric neurons express *Gr28a*.

### *Gr28a*-expressing neurons connect to IPCs

**Fig. 3.**
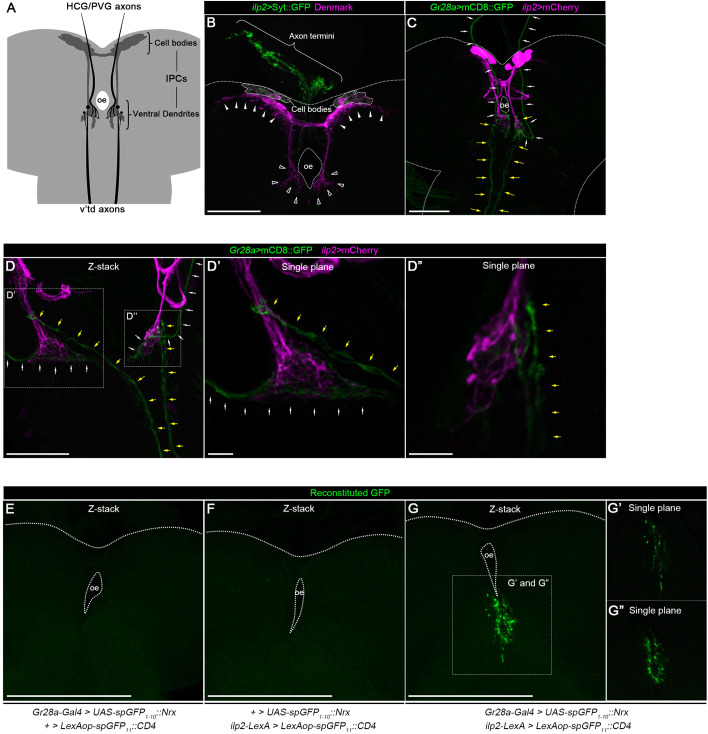
***Gr28a*-expressing neurons innervate to IPCs.** (A) Schematic of the axon termini of *Gr28a-Gal4*-positive v'td and HCG/PVG neurons projecting toward insulin-producing cells (IPCs) in the larval brain. (B) IPCs extend their dendrites to the protocerebrum and subesophageal zone (SEZ). Projection of confocal stacks of late second instar IPCs expressing GFP-fused synaptotagmin (Syt::GFP) (green) and a dendrite-localizing protein Denmark (magenta) under the control of *ilp2-Gal4* (*ilp2*>Syt::GFP, Denmark) is shown. Filled and open arrowheads indicate IPC dendrites in the protocerebrum and SEZ, respectively. Outlines of the brain and the cell bodies of IPCs are indicated by dotted and dashed lines, respectively. Axon termini of IPCs projecting to the aorta are indicated by a curly bracket. (C-D″) *Gr28a-Gal4*-positive neurons connect to IPCs in the SEZ. Projections (C,D) and sections (D′,D″) of confocal stacks of the late second instar CNS are shown. *Gr28a-Gal4* and *ilp2-LexA* expression was visualized by *UAS-mCD8::GFP* (green) and *LexAop-mCherry* (magenta), respectively. Yellow and white arrows indicate axon tracks of *Gr28a-Gal4*-positive v'td and HCG/PVG neurons, respectively. D′ and D″ show the magnified sections indicated by boxed areas in D. The brain is outlined by dotted lines. (E-G″) *Gr28a-Gal4*-positive neurons physically interact with IPCs in the SEZ. Projections (E-G) and sections (G′,G″) of confocal stacks of the late second instar CNS are shown. GFP signals from the reconstitution of spGFP_1-10_::Nrx and spGFP_11_::CD4 were observed in the animals carrying both *Gr28a-Gal4* and *ilp2-LexA*, which induce the expression of *UAS-spGFP_1-10_::Nrx* and *LexAop-spGFP_11_::CD4*, respectively (G–G″), but not in control animals carrying only *Gr28a-Gal4* (E) or *ilp2-LexA* (F) along with spGFP constructs. G′ and G″ show magnified sections of the boxed area in G. Note that reconstituted GFP signals were not observed in the lateral SEZ in which axon termini of *Gr28a-Gal4*-positive pharyngeal and external sensory neurons were detected (see Fig. 2K′). The brain is outlined by dotted lines. HCG, hypocerebral ganglion; oe, esophagus; PVG, proventricular ganglion. Scale bars: 100 µm (B-D,E-G); 10 µm (D′,D″).

Because the SEZ contains dendrites extended from protocerebral neuroendocrine cells including IPCs (Cao et al., 2014), we hypothesized that *Gr28a-*expressing neurons directly connect to the dendrites of IPCs in the SEZ. We confirmed that IPCs extend their dendrites not only to the protocerebrum but also to the SEZ (Fig. 3A,B). We next investigated the neuronal connection between *Gr28a-Gal4*-positive neurons and IPCs using *Gr28a-Gal4* and *ilp2-LexA*, which expresses LexA protein in IPCs (Li and Gong, 2015). *Gr28a-Gal4* and *ilp2-LexA* were labelled with *UAS-mCD8::GFP* and *LexAop-mCherry*, respectively. We found that the axon termini of *Gr28a-Gal4*-positive v'td and stomatogastric neurons are in close proximity to the IPC dendrites in the SEZ (Fig. 3C-D″). *Gr28a-Gal4*-positive stomatogastric neurons terminate on the posterior part of the IPC dendrites, whereas *Gr28a-Gal4*-positive v'td neurons extend their axon termini to the anterior part of the IPC dendrites (Fig. 3C-D″). The GFP Reconstitution Across Synaptic Partners (GRASP) analysis further indicates that *Gr28a-Gal4*-positive v'td neurons physically interact with IPCs: when membrane-tethered nonfunctional split-GFPs, *UAS-spGFP_1-10_::Nrx* and *LexAop-spGFP_11_::CD4*, were simultaneously expressed by *Gr28a-Gal4* and *ilp2-LexA*, respectively, reconstituted GFP signals were observed at two distinct SEZ loci that were identical to those innervated by v'td and stomatogastric *Gr28a-Gal4*-positive neurons (Fig. 3G-G″). By contrast, the reconstitution of GFP was not observed in control animals expressing either *Gr28a-Gal4* or *ilp2-LexA* alone along with spGFP constructs (Fig. 3E,F). These results indicate that both v'td and stomatogastric *Gr28a-Gal4*-positive neurons send signals to IPCs.

### *Gr28a*-expressing v'td neurons regulate larval development

**Fig. 4.**
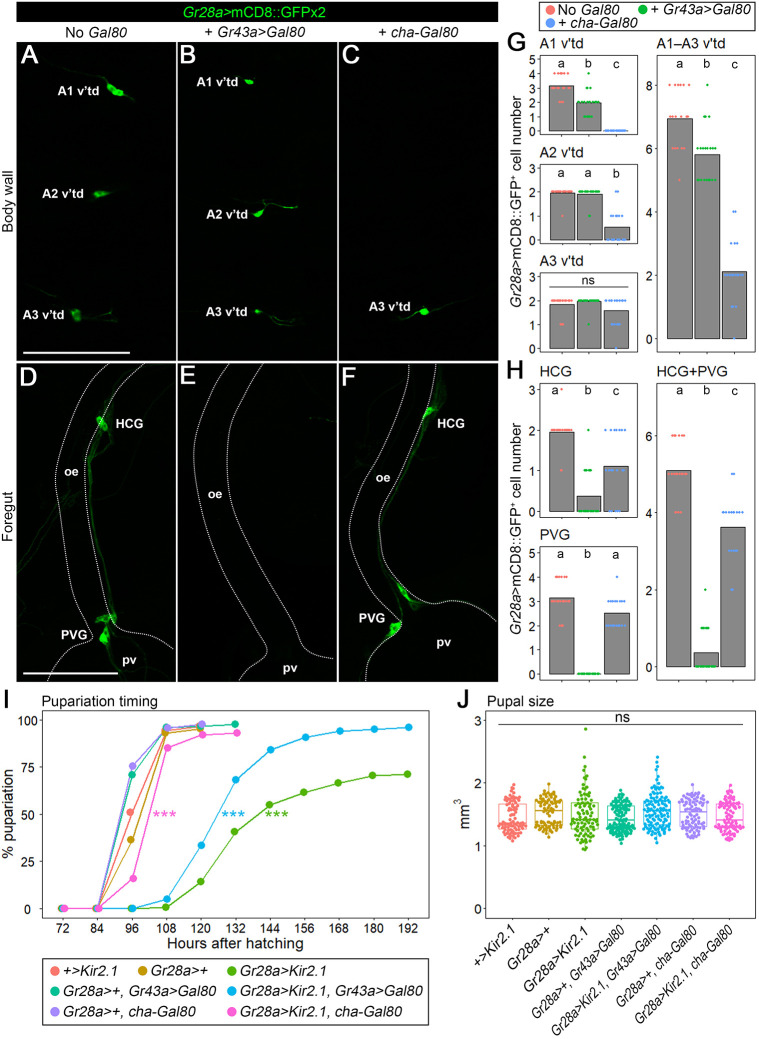
***Gr28a*-expressing v'td neuron activities are required for proper larval development.** (A-F) *Gr43a-LexA*-mediated expression of *Gal80* suppressed *Gr28a-Gal4* activity in HCG and PVG neurons, whereas *cha-Gal80* downregulated *Gr28a-Gal4* activity in v'td neurons. Confocal sections of the A1-A3 v'td neurons on the body wall (A-C) and projections of confocal stacks of HCG and PVG neurons on the foregut (D-F) in late second instar larvae are shown. *Gr28a-Gal4* activity visualized by two copies of *UAS-mCD8::GFP* (A,D) was suppressed either by *Gr43a-LexA* driving *LexAop-Gal80* (B,E) or *cha-Gal80* (C,F). The intestine is outlined by dotted lines in lower panels. (G,H) Numbers of the A1-A3 v'td neurons (G) and HCG/PVG neurons (H) expressing *Gr28a-Gal4*. Mean values are shown with jitter plots. Sample sizes are 18-22 for each genotype. Different lowercase letters indicate statistically significant differences (*P*<0.05; Steel-Dwass test). ns, not significant. (I) Silencing of *Gr28a-*expressing v'td neurons causes a delay in pupariation. Percentages of pupariated animals of each genotype at indicated time points are shown. Sample sizes are 85-135 for each genotype. ***P<0.001 (log-rank test, compared with *+>Kir2.1*). The raw *P*-values were multiplied by the number of independent tests (*n*=21) in accordance with the Bonferroni correction method (Jafari and Ansari-Pour, 2019). The full result of the statistical analysis is shown in Table S4. (J) Silencing of *Gr28a-*expressing v'td neurons does not cause an increase or a decrease in pupal size. Pupal sizes are shown in jitter and box plots. Box plots indicate median values and lower/upper quartiles, with whiskers extending to data points within 1.5 times the interquartile range. Sample sizes are 81-118 for each genotype. ns, not significant (*P*>0.05; Steel Dwass test). HCG, hypocerebral ganglion; oe, esophagus; pv, proventriculus; PVG, proventricular ganglion. Scale bars: 100 µm.

Next, we tested whether *Gr28a-Gal4* expression can be restricted by a Gal4 repressor Gal80 (Duffy, 2002). To suppress Gal4 expression in the stomatogastric neurons, *LexAop-Gal80* was expressed under the control of the *Gr43a* promoter using *Gr43a-LexA* (Miyamoto and Amrein, 2014), because *Gr43a-LexA* and *Gr28a-Gal4* are co-expressed in the HCG and PVG but not in v'td neurons (Fig. S4). We confirmed that *Gr43a>Gal80* blocked *Gr28a-Gal4* activity in the HCG/PVG clusters but had little effect on *Gr28a-Gal4* activity in v'td neurons (Fig. 4A,B,D,E). In contrast, *cha-Gal80*, a Gal80 transgene expressed in a cholinergic neuron-selective manner (Sakai et al., 2009), suppressed *Gr28a-Gal4* activity in the A1 and A2 v'td neurons, but it only slightly reduced the number of *Gr28a-Gal4*-positive HCG and PVG neurons (Fig. 4A,C,D,F). Overall, there were approximately seven *Gr28a-Gal4*-positive v'td neurons in control and six in *Gr43a>Gal80*-co-expressing animals, whereas *cha-Gal80* co-expression reduced it to two (Fig. 4G); conversely, the total number of *Gr28a-Gal4*-positive stomatogastric neurons was approximately five in control and four in *cha-Gal80*-co-expressing animals, whereas it was close to zero in *Gr43a>Gal80*-co-expressing larvae (Fig. 4H). In addition, both *Gr43a>Gal80* and *cha-Gal80* suppressed *Gr28a-Gal4* activity in the pharyngeal (VPS and PPS) and external (TOG and DOG) gustatory organs, as well as in the midgut (Fig. S5). Together, these results indicate that *Gr43a>Gal80* and *cha-Gal80* can restrict *Gr28a-Gal4* expression only to v'td and stomatogastric neurons, respectively.

Using these tools, we selectively silenced *Gr28a-*expressing v'td or stomatogastric neurons to investigate which *Gr28a-*expressing neuronal clusters are involved in the regulation of larval development. Control animals possessing either *UAS-Kir2.1* (*+>Kir2.1*) or *Gr28a-Gal4* (*Gr28a>+*) alone underwent pupariation between 84 and 108 hAH, whereas most *Gr28a>Kir2.1* animals pupariated after 108 hAH (Fig. 4I). Introduction of *Gr43a>Gal80* or *cha-Gal80* along with *Gr28a-Gal4* (*Gr28a>+, Gr43a>Gal80* and *Gr28a>+, cha-Gal80*) did not cause developmental delay (Fig. 4I). In contrast, *Gr28a>Kir2.1, Gr43a>Gal80* animals, in which the A1-A3 v'td neurons were selectively silenced, showed a significant delay in pupariation, with most animals pupariating after 108 hAH (Fig. 4I). On the other hand, *Gr28a>Kir2.1, cha-Gal80* animals, in which Kir2.1 was predominantly expressed in the HCG, PVG and A3 v'td neurons, only showed slight developmental delay (Fig. 4I). These results suggest that *Gr28a*-expressing A1 and A2 v'td neurons promote proper progression of larval development.

A previous study revealed that v'td neurons are labeled by not only *Gr28a-Gal4* but also *Gr28b.c-Gal4* and *Gr89a-Gal4* (Qian et al., 2018), which we also confirmed in both first and second instars (Fig. S2). Neuronal silencing using *Gr28b.c-Gal4* caused a significant developmental delay, which was slightly augmented when two copies of *Gr28b.c-Gal4* and *UAS-Kir2.1* were introduced (Fig. S2). Similarly, although *Gr89a-Gal4*-mediated silencing did not cause a significant developmental delay when a single copy of *UAS-Kir2.1* was used, introducing a second copy of *UAS-Kir2.1* caused a significant delay in pupariation (Fig. S2; note that homozygous *Gr89a-Gal4* animals are lethal). Overall, these results are consistent with the putative role of v'td neurons in promoting larval development, although the phenotypic severity is variable among these drivers, likely due to their differential Gal4 expression levels and patterns (Fig. S2). To further confirm the significance of v'td neuron functions during larval development, we therefore used two additional v'td neuron-selective Gal4 lines, *R73B01-Gal4* and *R22C07-Gal4* (Qian et al., 2018). *R73B01-Gal4* is expressed in v'td2 neurons throughout the body wall segment (Fig. S2) (Qian et al., 2018) and *R22C07-Gal4* is selectively expressed in A4-A6 v'td2 neurons (Fig. S2) (Qian et al., 2018). Silencing of *R73B01-Gal4*-expressing neurons (*R73B01>Kir2.1*), but not that of *R22C07*-expressing neurons (*R22C07>Kir2.1*), caused a significant delay in larval-prepupal transition to a similar extent as *Gr28a>Kir2.1* animals (Fig. S2). These results therefore further support the idea that A1-A3 v'td2 neuron activities are required for normal development. *R73B01>Kir2.1* animals also exhibited larval lethality, likely due to the driver's expression in other organs including the CNS, proventriculus and pharynx (Fig. S2). It is interesting to note that *Gr39a.b-Gal4*, which is highly expressed in the proventriculus (Fig. S1), also caused larval lethality when used to express *UAS-Kir2.1* in our screening (Fig. 1; Table S1).

As developmental delay potentially causes an increase in final body size (McBrayer et al., 2007), we measured the pupal size of *Gr28a-*expressing neuron-silenced animals and their controls. However, there was no significant difference in pupal size among the tested genotypes (Fig. 4J). It therefore seems that the growth rate is proportionally suppressed in *Gr28a>Kir2.1* and *Gr28a>Kir2.1, Gr43a>Gal80* animals.

We next sought to investigate the impact of v'td neuron-silencing on feeding behavior. As shown in Fig. S6, the v'td-silenced (*Gr28a>Kir2.1, Gr43a>Gal80*) and control (*+>Kir2.1* and *Gr28a>+, Gr43a>Gal80*) larvae cultured on a blue-colored medium were transferred to a red-colored medium at 36 hAH, and then the shift of consumed food color from blue to red (ratio of absorbance at 490 and 630 nm) was measured periodically to evaluate feeding efficiency. The result revealed that feeding is not significantly altered in the v'td-silenced larvae (Fig. S6C), suggesting that growth rate suppression is not caused by feeding defects in these animals.

### ILP release and larval growth are suppressed in *Gr28a*-expressing v'td neuron-silenced animals in a stage-specific manner

**Fig. 5.**
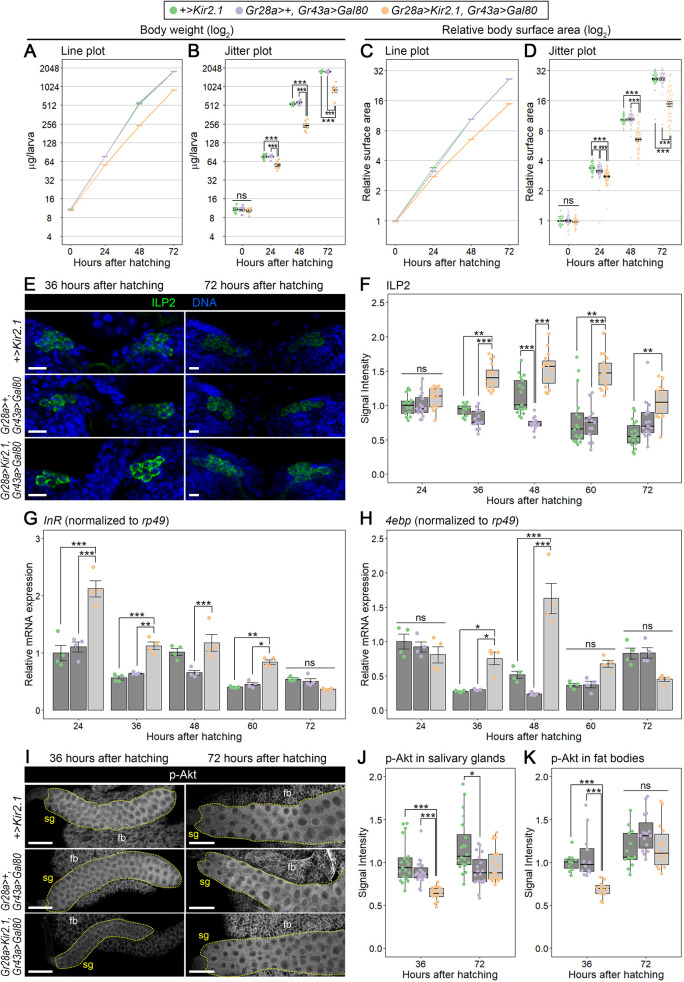
***Gr28a*-expressing v'td neurons promote ILP secretion and growth in a stage-specific manner.** (A-D) Silencing of *Gr28a-*expressing v'td neurons causes growth retardation. Body weight (A,B) and relative body surface area (C,D) of control (*+>Kir2.1* and *Gr28a>+, Gr43a>Gal80*) and *Gr28a*-expressing v'td neuron-silenced (*Gr28a>Kir2.1, Gr43a>Gal80*) larvae at indicted time points are shown. In A and C, line plots with mean values (bold horizontal lines) are shown for each genotype. In B and D, mean values (bold black horizontal lines) with s.e.m. are shown in jitter plots. In A, sample sizes are 7 in all groups (8-20 larvae were pooled and body weight per larva was calculated for each sample). In B, sample sizes are 34-57 in each group. Statistical analysis using Tukey's test was performed for each time point. Asterisks show statistically significant differences (*P<0.05; ***P<0.001). (E) Silencing of *Gr28a-*expressing v'td neurons causes accumulation of ILP2 in insulin-producing cells (IPCs). Confocal sections of the larval brains of control and *Gr28a*-expressing v'td neuron-silenced animals stained with anti-ILP2 antibody (green) and Hoechst (blue) at 36 hAH (left panels) and 72 hAH (right panels) are shown. (F) The ILP2 signal intensities in IPCs of control and *Gr28a*-expressing v'td neuron-silenced larvae at indicated time points. Data are shown in jitter and box plots. Box plots indicate median values and lower/upper quartiles, with whiskers extending to data points within 1.5 times the interquartile range. Sample sizes are 11-18 in each group. Statistical analyses using Steel-Dwass test were performed for all groups (**P<0.01; ***P<0.001). ns, not significant (*P*>0.05). The full result of the statistical analysis is shown in Table S4. (G,H) Silencing of *Gr28a*-expressing v'td neurons causes upregulation of *InR* and *4ebp* gene expression. Relative expression levels of *InR* (G) and *4ebp* (H) in control and *Gr28a*-expressing v'td neuron-silenced larvae at indicated time points are shown. Expression levels of target genes were normalized to *rp49* expression levels. Mean values of quadruplicated datasets are shown as bar graphs with s.e.m. and jitter plots. Statistical analyses using Tukey's test were performed for all groups. *P<0.05; **P<0.01; ***P<0.001. ns, not significant (*P*>0.05). The full result of the statistical analysis is shown in Table S4. (I) Silencing of *Gr28a*-expressing v'td neurons causes downregulation of Akt phosphorylation at 36 hAH. Confocal sections of the larval salivary glands (sg) and fat bodies (fb) of control and *Gr28a*-expressing v'td neuron-silenced animals stained with anti-p-Akt antibody at 36 hAH (left panels) and 72 hAH (right panels) are shown. Dotted line outlines the salivary gland. (J,K) The p-Akt signal intensities in salivary glands (J) and fat bodies (K) of control and *Gr28a*-expressing v'td neuron-silenced larvae at 36 and 72 hAH. Data are shown in jitter and box plots. Box plots indicate median values and lower/upper quartiles, with whiskers extending to data points within 1.5 times the interquartile range. Sample sizes are 15-20 (salivary glands) and 11-15 (fat bodies) in each group. Statistical analyses using Steel-Dwass test were performed for all groups in each tissue. *P<0.05; ***P<0.001. ns, not significant (*P*>0.05). The full result of the statistical analysis is shown in Table S4. Scale bars: 10 µm (E); 100 µm (I).

To further investigate the potential growth defect, we next measured the body size of the v'td-silenced (*Gr28a>Kir2.1, Gr43a>Gal80*) and control (*+>Kir2.1* and *Gr28a>+, Gr43a>Gal80*) animals periodically during larval development. The body surface area and body weight were only slightly reduced in the v'td-silenced larvae compared with control animals at 24 hAH (Fig. 5A-D). In contrast, the v'td-silenced larvae were significantly smaller at 48 and 72 hAH (Fig. 5A-D), indicating that larval growth is suppressed in the v'td-silenced animals after 24 hAH. As evident in line plots (Fig. 5A,C), growth rate (i.e. slope of the growth curve) was relatively low in the v'td-silenced animals between 24 and 48 hAH, whereas it was equivalent to control from 48 to 72 hAH. These results indicate that growth is predominantly downregulated from 24 to 48 hAH in the v'td-silenced animals.

Interestingly, homozygous *Gr28a^GAL4^* mutant larvae (*Gr28a^GAL4^/Gr28a^GAL4^*) also had lower body weight compared with wild type (*+/+*) and heterozygous *Gr28a^GAL4^* mutants (*Gr28a^GAL4^/+*) at 48 and 72 hAH, but not at 24 hAH (Fig. S3E,F). As indicated in the line plot (Fig. S3E), growth rate between 24 and 48 hAH was reduced in homozygous *Gr28a^GAL4^* larvae, whereas the growth rate between 48 and 72 hAH was equivalent to wild-type and heterozygous *Gr28a^GAL4^* animals. In addition, there was no significant difference in the pupal size between wild-type, heterozygous *Gr28a^GAL4^* and homozygous *Gr28a^GAL4^* animals (Fig. S3H). These results indicate that the stage-specific growth-promoting effect of *Gr28a-*expressing v'td neurons from 24 to 48 hAH may indeed be mediated by Gr28a. However, developmental delay phenotype observed in homozygous *Gr28a^GAL4^* mutant animals (Fig. S3G) was milder than *Gr28a-*expressing v'td neuron-silenced animals (Fig. 4I), suggesting that the v'td neuron activity is not completely suppressed in *Gr28a* mutants. On the other hand, continuous activation of *Gr28a-*expressing v'td neurons by forced expression of a sodium channel NaChBac (Nitabach et al., 2006) caused neither acceleration of larval growth nor an increase in pupal volume (Fig. S7). These results suggest that the v'td neuron activity is not sufficient for promoting larval growth and further support the idea that their activity is important during a specific stage of development.

The above observations raised the possibility that ILP secretion is temporarily downregulated in the v'td-silenced animals during larval development. To test this, we performed immunostaining to quantify the accumulated amount of ILP2, one of the major ILPs produced in IPCs. In accordance with the growth phenotype, there was no significant difference in ILP2 immunostaining levels among controls and the v'td-silenced animals at 24 hAH, whereas the ILP2 signal intensity was significantly elevated in the latter at 36, 48 and 60 hAH (Fig. 5E,F), indicating that IPC activity was downregulated in the v'td-silenced animals at these time points. The ILP2 signal intensity was still higher in the v'td-silenced animals against one control at 72 hAH (Fig. 5F). However, considering that the signal intensity was decreased from 60 to 72 hAH in these larvae (Fig. 5F), IPC activity was likely restored by 72 hAH in the v'td-silenced animals. As *ilp2* gene expression levels were not increased in the CNS of the v'td-silenced animals at 36 and 72 hAH (Fig. S8A-C), the increase of ILP2 immunostaining intensity in IPCs of the v'td-silenced larvae is most likely due to impaired ILP2 secretion but not *ilp2* transcriptional upregulation.

We further sought to investigate whether insulin signaling activity was decreased in the v'td-silenced animals. Transcript levels of *insulin-like receptor* (*InR*) and *eIF-4E-binding protein* (*4ebp*/*Thor*), expression of which is suppressed by insulin signaling (Hietakangas and Cohen, 2009), were elevated in the v'td-silenced animals at 36 and 48 hAH, but not at 72 hAH (Fig. 5G,H; Fig. S8). Furthermore, protein levels of the phosphorylated form of Akt (p-Akt), a protein kinase activated in an insulin signaling-dependent manner (Hietakangas and Cohen, 2009), were decreased in the v'td-silenced larvae at 36 hAH, whereas they were equivalent to control animals at 72 hAH (Fig. 5I-K). Taken together, these results indicate that ILP release and subsequent insulin signaling are upregulated by *Gr28a-*expressing v'td neurons predominantly during the mid-larval period (i.e. between 24 and 72 hAH), although the small reduction in body size (Fig. 5A-D) and an increase in *InR* expression levels (Fig. 5G; Fig. S8D) at 24 hAH in the v'td-silenced animals may suggest potential functions of these neurons at even earlier stages.

## DISCUSSION

Grs are a group of transmembrane chemosensory receptors expressed in external and pharyngeal gustatory neurons as well as in internal sensory neurons. They have a central role in sensation of various environmental cues, including nutrients and noxious compounds, to regulate feeding behavior. However, the importance of Gr-expressing neurons in regulating larval growth remains mostly unclear. In this study, we screened 66 *Gr-Gal4* lines using a neural silencer *UAS-Kir2.1* to identify GRNs regulating larval growth. Notably, most *Gr-Gal4* lines expressed in various populations of GRNs did not induce any major developmental defect (Fig. 1; Table S1). Gr-expressing gustatory neurons thus seem to be mostly dispensable for larval development in standard lab conditions. Alternatively, it is possible that GRNs can compensate for loss of other functionally analogous GRNs during development.

Here, we identified *Gr28a*-expressing internal sensory neurons as a novel regulator of larval development. Our results suggest that *Gr28a*-expressing v'td neurons regulate IPC activity and thereby control growth rate mainly between 24 and 72 hAH (Fig. 5). At around 60 hAH, *Drosophila* larvae attain the minimum body weight required for initiation of metamorphosis in normal schedule, which is known as the ‘critical weight checkpoint’ (Mirth et al., 2005; Ohhara et al., 2017). Considering that the attainment of critical weight is promoted by insulin signaling (Koyama et al., 2014; Mirth et al., 2005; Ohhara et al., 2017), one possibility is that *Gr28a*-expressing v'td neurons are involved in the attainment of critical weight through regulation of ILP secretion. Although regulatory mechanisms of ILP secretion by v'td neurons remain unclear, we speculate that *Gr28a*-expressing v'td neurons innervate IPCs to regulate their responsiveness to insulinotropic/insulinostatic signaling pathways, including fat body-derived endocrine signals during this growth period.

An important unanswered question is what types of sensory inputs are received by *Gr28a*-expressing v'td neurons. Their dendrites are associated with tracheal branches and exposed to the hemolymph (Qian et al., 2018), suggesting that these neurons may respond to humoral cues. Considering that a developmental delay observed in *Gr28a* mutant animals was milder than that of the v'td neuron-silenced animals (Fig. 4I; Fig. S3G), it is most likely that v'td neurons sense humoral cues not only via Gr28a but also through other receptors such as Gr28b that are known to be expressed in v'td neurons (Qian et al., 2018). It has been reported that Gr28a and Gr28b mediate RNA sensing and that Gr28a is also required for sensing ribonucleosides including uridine and inosine (Mishra et al., 2018a). Thus, one possibility is that *Gr28a*-expressing v'td neurons regulate larval growth in accordance with the amount of RNA and ribonucleosides diffused in the hemolymph. Interestingly, depletion of extracellular adenosine by administration of extracellular adenosine deaminase, an enzyme converting adenosine to inosine, promotes cell proliferation *in vitro* (Zurovec et al., 2002), suggesting that extracellular adenosine inhibits proliferation. Furthermore, loss of adenosine deaminase causes a delay in pupariation (Dolezal et al., 2005). It is therefore conceivable that *Gr28a*-expressing v'td neurons monitor inosine concentration in the hemolymph to control insulin signaling and growth rate in accordance with inosine availability. In addition, a recent study has reported that Gr28b.c, which is co-expressed with Gr28a in v'td neurons (Qian et al., 2018), mediates sensation of plant-derived saponin, an amphipathic glycoside, in external sensory organs in the adult stage (Sang et al., 2019), although whether dietary saponin is incorporated into hemolymph is unknown. Further studies are required to elucidate sensory cues affecting the activity of *Gr28a*-expressing v'td neurons and their biological significance.

**Fig. 6.**
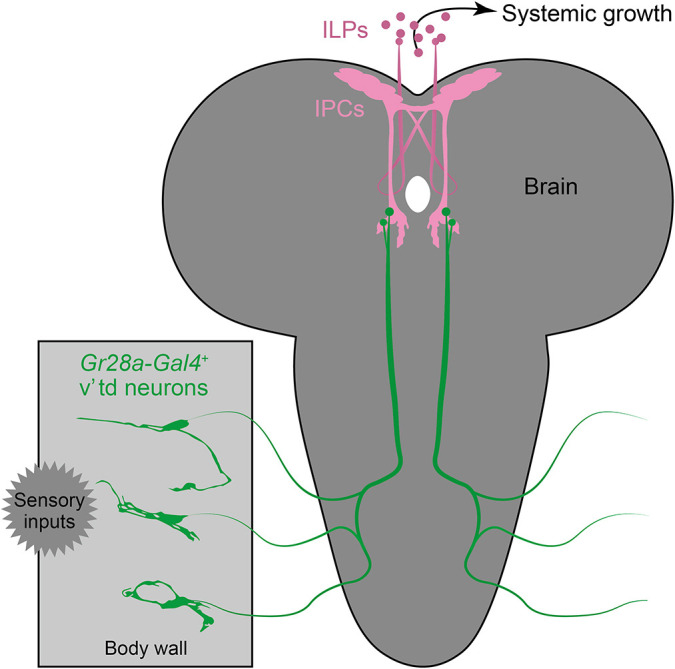
**A model for *Gr28a-*expressing v'td neuron-mediated regulation of larval growth.** Body wall-associated *Gr28a-*expressing v'td neurons, which are potentially receiving sensory input(s), directly project to the insulin-producing cells (IPCs) to stimulate ILP release and systemic growth.

In summary, we identified body wall-associated *Gr28a* neurons as stage-specific insulinotropic sensory neurons (Fig. 6). To the best of our knowledge, GRNs regulating ILP release and systemic growth have not been reported previously. This study thus provides an important basis to further elucidate neuroendocrine pathways regulating insect growth and development.

## MATERIALS AND METHODS

### *Drosophila* stocks and media

Fly stocks and their genotypes used in this study are summarized in Tables S2 and S3. *Gr-Gal4* lines used in our screening are listed in Table S1. Fly stocks were maintained on a standard *Drosophila* cornmeal/sucrose/yeast medium supplemented with 0.3% (vol/vol) propionic acid and 0.35% (vol/vol) butylparaben (167 mg/ml in 70% ethanol) at 25°C under a 12 h light/12 h dark cycle. For *Gr-Gal4* developmental timing screening, flies were reared on a standard fly food described in Okamoto et al. (2018). A nutrient-rich semi-defined medium called German formulation (Backhaus et al., 1984) was used to investigate developmental profiles, body size and gene expression in *Gr28a* neuron-silenced animals. We added 22.5 g of German formulation powder (Genesee Scientific, 66-115) to 100 ml of water and boiled using a microwave. After stirring for 30 min at room temperature, 600 µl of propionic acid was added to obtain the German formulation medium.

### Generation of a *Gr28a* mutant strain

*Gr28a* was deleted and replaced with a Gal4-3xP3-RFP cassette using CRISPR/Cas9-mediated genome editing by WellGenetics as follows. In brief, a Gal4-3xP3-RFP cassette, which contains a ribosome binding sequence, Gal4, SV40 3′ untranslated region (UTR), α-Tubulin 3′UTR, a floxed 3xP3-RFP and two homology arms were cloned into *pUC57-Kan* as a donor template for repair. *Gr28a*-targeting gRNAs (upstream: CGGAAAATACGCGAGTCGAG[CGG]; downstream: TACGTCACTGACTGTGTTGC[TGG]) and hs-Cas9 were supplied in DNA plasmids together with the donor plasmid for microinjection into embryos of *w^1118^* to delete a 1929 bp fragment of *Gr28a* (−57 nt to +1872 nt from the start codon) and replace the deleted region with a Gal4-3xP3-RFP cassette by homology dependent repair.

### Developmental staging and larval body weight measurement

Parent flies were maintained in plastic bottles (Genesee Scientific, 32-310) and allowed to lay eggs for 24 h on grape juice agar plates (2 g agar in 100 ml grape juice, poured into a 4.5 cm×1.6 cm plastic dish) supplemented with dry yeast powder (Oriental Yeast Co.). Newly hatched larvae were transferred to vials containing the culture medium described above. Larvae were cultured at 25°C under a 12 h light/12 h dark cycle, and developmental stages and lethality were scored every 12 h.

To measure larval body weight, larvae were washed with distilled water, blotted on Kimwipes (Crecia) and collected in 1.5 ml plastic tubes before weighing. Larvae were weighed in more than three batches of 8-20 larvae in each experimental group. Body weight of individual larvae was then calculated for each batch.

### Pupal volume measurement

Pupae were aligned on a slide glass and images were acquired using an Olympus SZX10 stereomicroscope with an Olympus DP26 CCD camera. Length and width of pupae were measured using Image J/Fiji software (Schindelin et al., 2012) and pupal volume was determined using the following calculation: 4/3 π (L/2) (l/2)^2^ (L, length; l, width).

### Feeding behavior assay

Larvae were cultured on a German formulation medium with 0.1% (wt/wt) Blue No.1 (F0147, Tokyo Chemical Industry) until 36 hAH. Larvae were washed three times with distilled water to remove the blue-colored medium and then transferred to a German formulation medium with 0.2% (wt/wt) Red No.40 (A0943, Tokyo Chemical Industry). Larvae were sampled at 0, 30, 60 and 120 min after transfer and stored at −80°C. Frozen larvae were homogenized in 55 µl of 0.1% PBT [0.01% Triton X-100 in phosphate buffered saline (PBS)], centrifuged at 12,000 ***g*** for 10 min, and the supernatant was transferred to a 96-well microplate (Sumitomo Bakelite). Absorbance at 490 and 630 nm in the extracts was measured using the microplate reader (SpectraMax 190, Molecular Devices), and the ratio of absorbance at 490 and 630 nm was calculated.

### Quantitative RT-PCR (qPCR)

qPCR was performed to measure the expression levels of *InR*, *4ebp* and *ilp2*. Total RNA was extracted from larvae using TRIzol (Thermo Fisher Scientific, 15596026). For RNA extraction from larval brains, larvae were dissected in PBS to collect brains and then total RNA was extracted from pooled brains (four brains/sample) using the RNeasy Micro kit (Qiagen, 74004). Reverse transcription was performed using the SuperScript III kit (Thermo Fisher Scientific, 18080051), and the obtained cDNA was used as a template for qPCR using the Quantifast SYBR Green PCR kit (Qiagen, 204056) and Rotor-Gene Q (Qiagen). All reactions were performed at 95°C for 10 min, followed by 50 cycles of 95°C for 10 s and 60°C for 30 s. Dissociation curve analysis was applied to all reactions to ensure the presence of a single PCR product. Expression levels of the target gene were calculated using the relative standard curve method (Larionov et al., 2005). Stock cDNA used for the relative standard curves was synthesized from pooled RNA derived from larvae reared under the same conditions and diluted serially. Expression levels of the target gene were normalized to the endogenous reference genes *ribosomal protein 49* (*rp49*; also known as *ribosomal protein L32*) and *ribosomal protein l23* (*rpl23*). The mean expression level of the control at 24 hAH (whole body) or 36 hAH (brain) was set to 1. The primer sets used for qPCR are as follows (5′ to 3′): *rp49* forward, ACAAATGGCGCAAGCCCAAGG; *rp49* reverse, ATGTGGCGGGTGCGCTTGTT; *rpl23* forward, GACAACACCGGAGCCAAGAACC; *rpl23* reverse, GTTTGCGCTGCCGAATAACCAC; *InR* forward, CGATTTCACGGAAGTCGAAC; *InR* reverse, GAACAGGGAAACGATCAGGA; *4ebp* forward, CCATGATCACCAGGAAGGTTGTCA; *4ebp* reverse, AGCCCGCTCGTAGATAAGTTTGGT; *ilp2* forward, ACGAGGTGCTGAGTATGGTGTGCG; *ilp2* reverse, CACTTCGCAGCGGTTCCGATATCG.

### Immunohistochemistry and histochemistry

Immunostaining was performed to visualize ILP2 in IPCs, observe neurons and tissues expressing GFP or mCherry (mCherry was observed without signal enhancement), and measure the level of phosphorylated Akt kinase (p-Akt). Larvae were dissected in PBS and fixed for 25 min with 4% paraformaldehyde in 0.01% PBT. Tissues were washed with 0.1% PBT three times for 10 min each and washed with 1% PBT for 5 min to increase antibody permeability. Tissues were then blocked with 1% goat serum (Sigma-Aldrich, G9023) in 0.1% PBT for 30 min, and incubated at 4°C overnight with a primary antibody in the blocking solution at the following dilution rates: anti-ILP2 at 1:1000 (rabbit polyclonal, a gift from T. Nishimura; Okamoto et al., 2012), anti-GFP at 1:1000 (mouse monoclonal, Thermo Fisher Scientific, A11120), anti-Bruchpilot (Brp) at 1:50 (Developmental Studies Hybridoma Bank, nc82) or anti-p-Akt at 1:500 (rabbit polyclonal, Cell Signaling Technology, 4054). Note that GFP signals were not enhanced by anti-GFP antibody for Brp staining in *Gr28a>mCD8::GFPx2* animals. Tissues were washed with 0.1% PBT three times for 10 min each and incubated at 4°C overnight with Alexa 488-conjugated goat anti-rabbit IgG (Thermo Fisher Scientific, A11008), Alexa 488-conjugated goat anti-mouse IgG (Thermo Fisher Scientific, A11001) or Alexa 546-conjugated goat anti-mouse IgG (Thermo Fisher Scientific, A11003) at 1:1000 dilution in 0.1% PBT. For ILP2 staining, Hoechst 33258 (Thermo Fisher Scientific, H3569) was used at 1:1500 dilution with the secondary antibody to detect double-strand DNA. For observation of *Gr39a.b-Gal4*-driven GFP in the intestine, Alexa 546-conjugated phalloidin (Thermo Fisher Scientific, A22283) was used at 1:500 dilution with the secondary antibody in 0.1% PBT. After washing with 0.1% PBT three times for 10 min each, tissues were dissected and mounted in a mounting medium. Images were taken with a Zeiss LSM700 or Zeiss Axio Imager M2 equipped with ApoTome.2. Confocal *z*-series were obtained using a 1 µm step size and identical laser power and camera gain settings.

### GRASP

In GRASP analysis, two nonfunctional split-GFP constructs, *UAS-spGFP_1-10_::Nrx* and *LexA-spGFP_11_::CD4*, were expressed on the cell membrane of two distinct neuronal clusters by *Gr28a-Gal4* and *ilp2-LexA*, respectively (Tables S2 and S3). The presence of reconstituted GFP signal was assessed as follows. Larvae were dissected in PBS and fixed for 25 min with 4% paraformaldehyde in 0.01% PBT. After washing with 0.1% PBT three times for 10 min each, tissues were dissected and mounted in a mounting medium. Images were taken with a Zeiss LSM700 using the same settings as described in ‘Immunohistochemistry and histochemistry’.

### Image analyses

To quantify ILP2 immunostaining signal intensity, confocal *z*-series were processed using Image J/Fiji software (Schindelin et al., 2012). First, a series of sections was maximal-intensity *z*-projected, and the *z*-projected image was duplicated. Next, ILP2 signal in either of *z*-projected images was binarized, and then the binarized area was selected as a region of interest (ROI) using the ‘Analyzed particles’ function. ROI were selected in a non-binarized *z*-projected image, and the mean value of ILP2 signal intensity within the ROI was measured.

Quantification of p-Akt immunostaining signal intensity was performed as follows. A series of sections were maximal intensity *z*-projected, and the mean values of immunostaining signal intensity in salivary glands and fat bodies were measured within the ROI that was selected manually by freehand selection.

### Statistical analyses

Statistical analyses were performed using R version 3.6.2 (http://www.R-project.org/) (Ihaka and Gentleman, 1996). Data were analyzed using different tests as indicated in figure legends.

## Supplementary Material

10.1242/develop.200440_sup1 Supplementary information
