# Supplementary material for: Internal sensory neurons regulate stage-specific growth in Drosophila
Source: Development. 2022 Oct 28;149(21):dev200440. doi: 10.1242/dev.200440 (PMC10496149; doi:10.1242/dev.200440)
Supplement: Supplementary information [file develop-149-200440-s1.pdf]

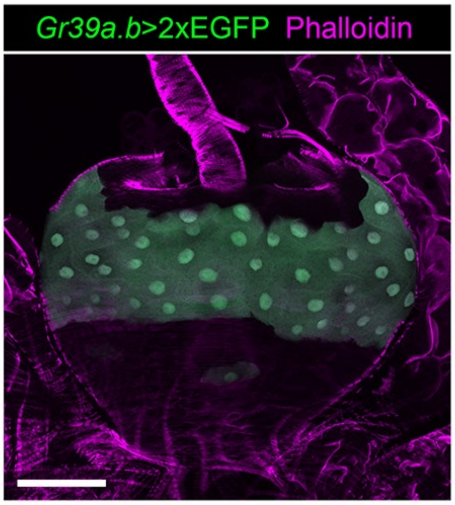

Fig. S1. Expression pattern of *Gr39a.b-Gal4* in the larval intestine.

A confocal section of the proventriculus of a late 2nd instar larva expressing *Gr39a.b-Gal4*. *Gr39a.b-Gal4* expression was visualized by *UAS-2xEGFP* (green). F-actin was stained by phalloidin (magenta). Scale bar = 100 μm.

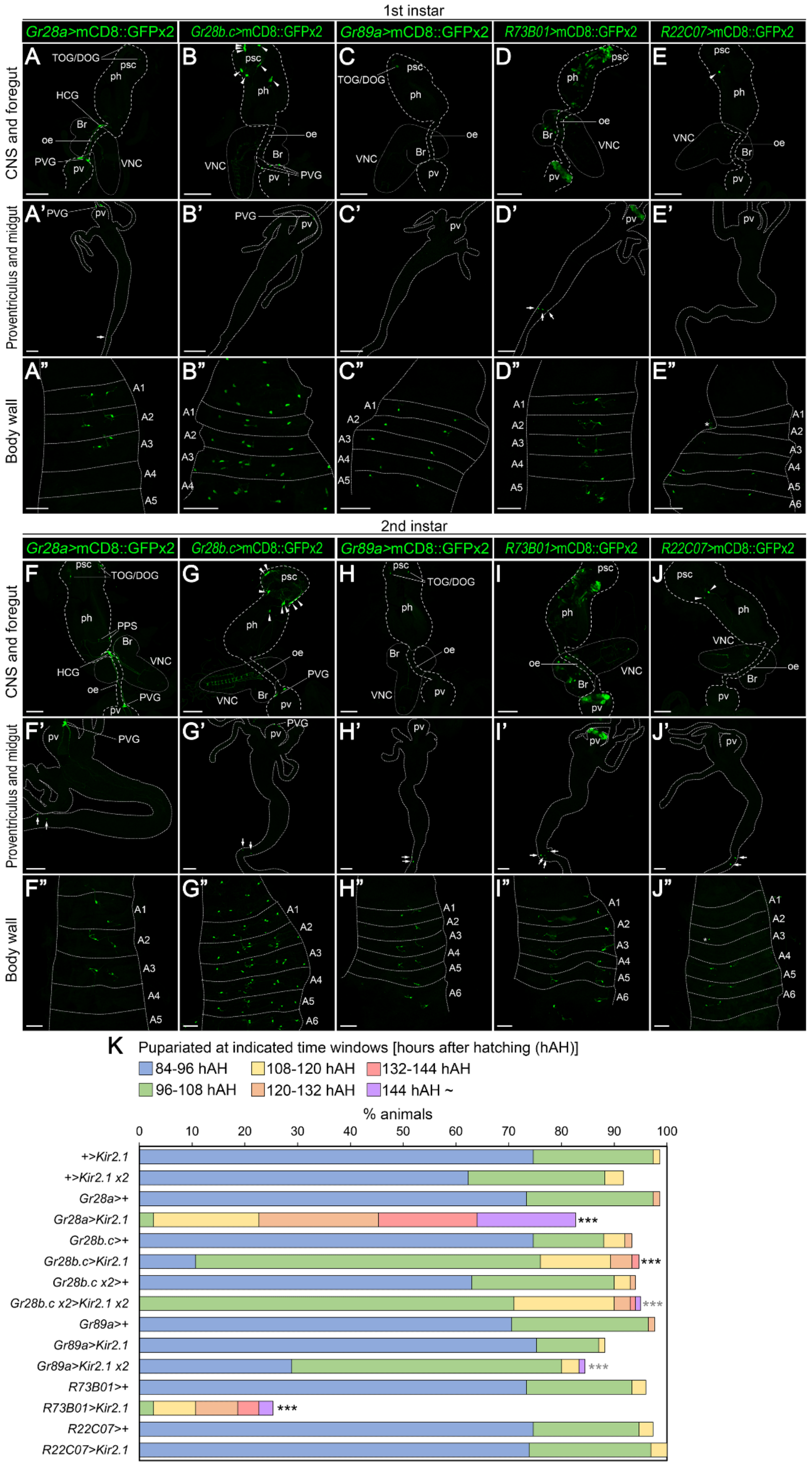

Development • Supplementary information

Fig. S2. Expression patterns of Gal4 lines that label v’td neurons and developmental phenotype of v’td neuron-silenced animals.

(A–J”) Projections of confocal stacks of the CNS and foregut (A–J), proventriculus and midgut (A’–J’), and body wall (A”–J”) of 1st and 2nd instar larvae expressing *Gr28a-Gal4* (A –A” and F–F”), *Gr28b.c-Gal4* (B–B” and G–G”), *Gr89a-Gal4* (C–C” and H–H”), *R73B01-Gal4* (D–D” and I–I”), and *R22C07-Gal4* (E–E” and J–J”) as visualized by two copies of *UAS-mCD8::GFP*. Outlines of the CNS and pharynx/intestine are indicated by dotted and dashed lines, respectively (A–J and A’–J’). Dotted lines in A”–J” indicate segment borders of the body wall. Arrows indicate GFP-positive midgut cells. Arrowheads indicate unidentified pharyngeal neurons. Scale bars = 100 μm. Br, brain; psc, pseudocephalon; ph, pharynx; oe, oesophagus; pv, proventriculus. **(K)** Percentages of control (*+>Kir2.1*, *+>Kir2.1 x2*, *Gr28a>+*, *Gr28b.c>+*, *Gr28b.c x2>+*, *Gr89a>+*, *R73B01>+*, and *R22C07>+*) and v’td neuron-silenced larvae (*Gr28a>Kir2.1*, *Gr28b.c>Kir2.1*, *Gr28b.c x2>Kir2.1 x2*, *Gr89a>Kir2.1*, *Gr89a>Kir2.1 x2*, *R73B01>Kir2.1*, and *R22C07>Kir2.1*) pupariated within the indicated time window. Sample sizes are 65–100 for each genotype. Asterisks indicate statistically significant differences [****P* < 0.001; log-rank test as compared to *+>Kir2.1* (black) or *+>Kir2.1 x2* (gray)]. The result of the statistical analysis is shown in Table S4.

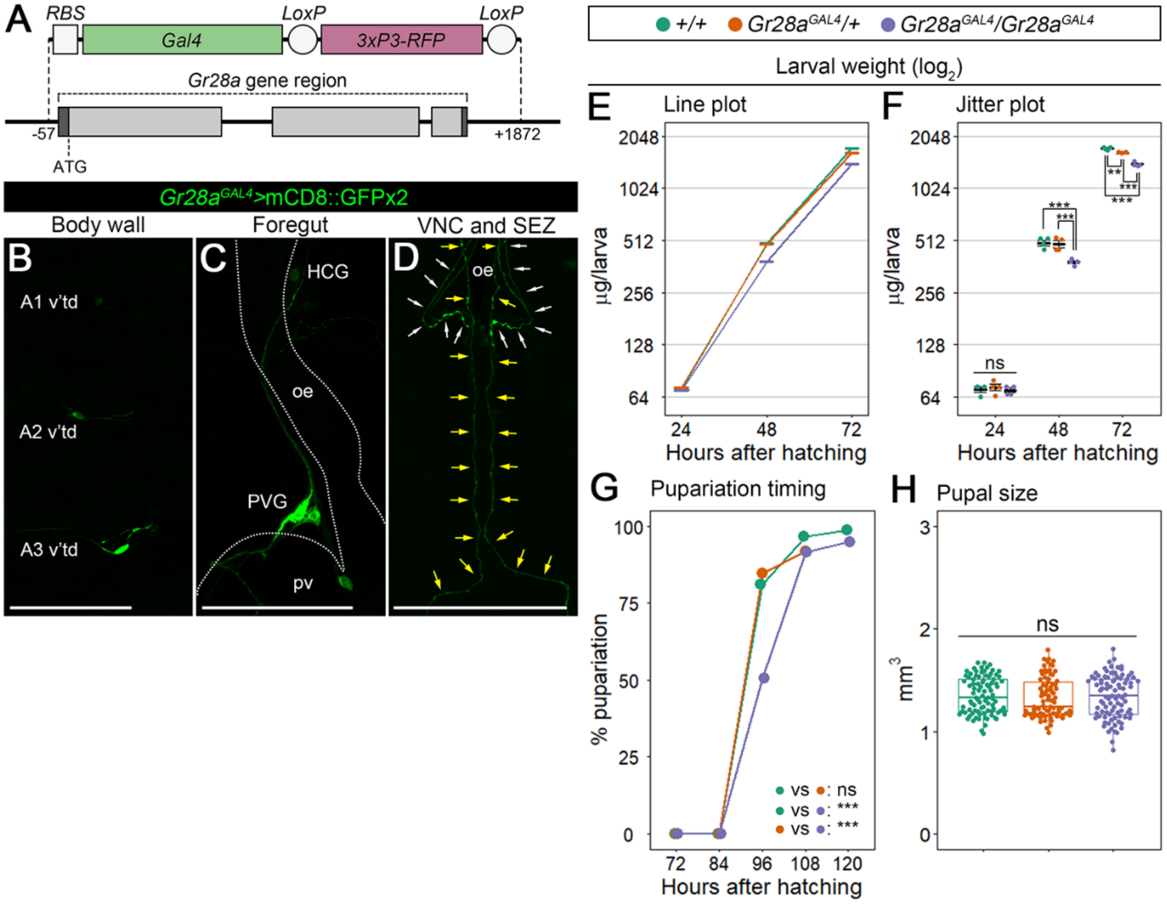

Fig. S3. Characterization of a *Gr28a*^{GAL4} strain.

(A) Schematic diagram of the *Gr28a* gene locus and a Gal4-3xP-RFP cassette that was used to replace the Gr28a gene to generate a *Gr28a*^{GAL4} mutant strain. A 1929-bp fragment of *Gr28a* (-57 nt to +1,872 nt from the start codon) was deleted by two gRNAs and replaced with a Gal4-3xP-RFP cassette with *LoxP* sites. Dark and light gray boxes show UTR and coding regions of *Gr28a*, respectively. RBS, ribosome binding sequence. **(B–D)** Projections of confocal stacks of A1–A3 v'td neurons on the body wall (B), HCG and PVG neurons on the foregut (C), and the VNC and SEZ (D) in late 2nd instar *Gr28a*^{GAL4} larvae expressing two copies of *UAS-mCD8::GFP*. The intestine is outlined by dotted lines. Yellow and white arrows indicate axon tracks of *Gr28a-Gal4*-positive v'td and HCG/PVG neurons, respectively. oe, oesophagus; pv, proventriculus. Scale bars = 100 μm. **(E and F)** Body weight of wild type (+/+) and heterozygous and homozygous *Gr28a*^{GAL4} mutants (*Gr28a*^{GAL4}/+ and *Gr28a*^{GAL4}/*Gr28a*^{GAL4}, respectively) at indicted time points. In E, line plots with average values (bold lines) are shown for each genotype. In F, average values (bold black lines) with SE are shown in jitter plots. Sample sizes are 4 in all groups (6–20 larvae were pooled and body weight per larva was calculated for each sample). Statistical analyses using Tukey's test were performed for each time point. Asterisks indicate significant differences (**$P < 0.01$; ***$P < 0.001$). ns, not significant ($P > 0.05$). **(G)** Percentages of pupariated animals of each genotype at indicated time points. Sample sizes are 85–95 for each genotype. Results of statistical tests using the log-rank test are shown in the lower right (***$P < 0.001$). ns, not significant ($P > 0.05$). Raw *P*-values were multiplied by the number of independent tests (m = 3) in accordance with the Bonferroni correction method (Jafari and Ansari-Pour, 2019). **(H)** Pupal sizes of each genotype as shown in jitter and box plots. Sample sizes are 78–90 for each genotype. ns, not significant ($P > 0.05$; Steel-Dwass test).

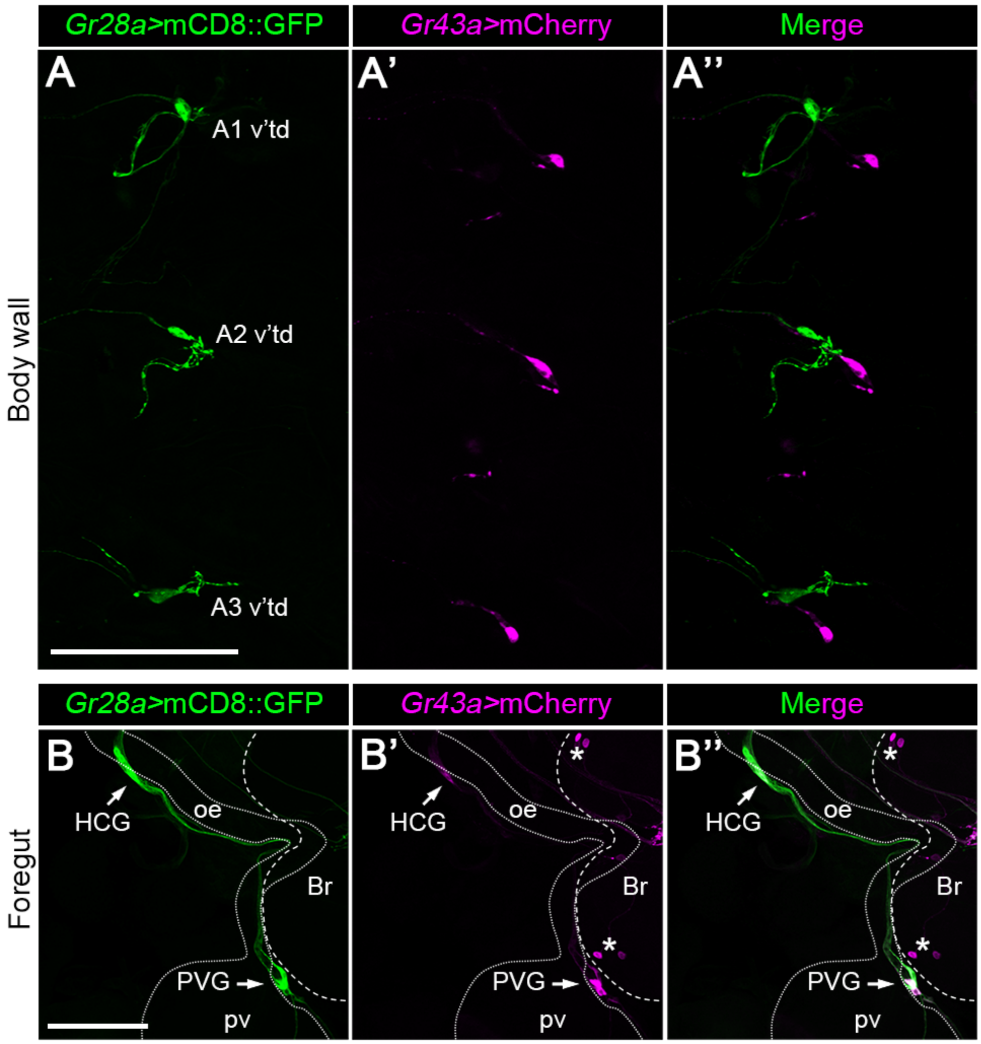

Fig. S4. Expression patterns of *Gr28a-Gal4* and *Gr43a-LexA* in body wall-associated and stomatogastric neurons.

Projections of confocal stacks of v'td neurons (A–A'') and HCG and PVG neurons (B–B'') in late 2nd instar larvae expressing *Gr28a-Gal4* and *Gr43a-LexA*. *Gr28a-Gal4* and *Gr43a-LexA* expression was visualized by *UAS-mCD8::GFP* (green) and *LexAop-mCherry* (magenta), respectively. Outlines of the brain (Br) and the intestine including oesophagus (oe) and proventriculus (pv) are indicated by dashed and dotted lines, respectively. Arrows indicate *Gr28a-Gal4*-positive HCG and PVG neurons. Asterisks indicate *Gr43a-LexA*-positive neurons in the brain. Scale bars = 100 μm.

Development • Supplementary information

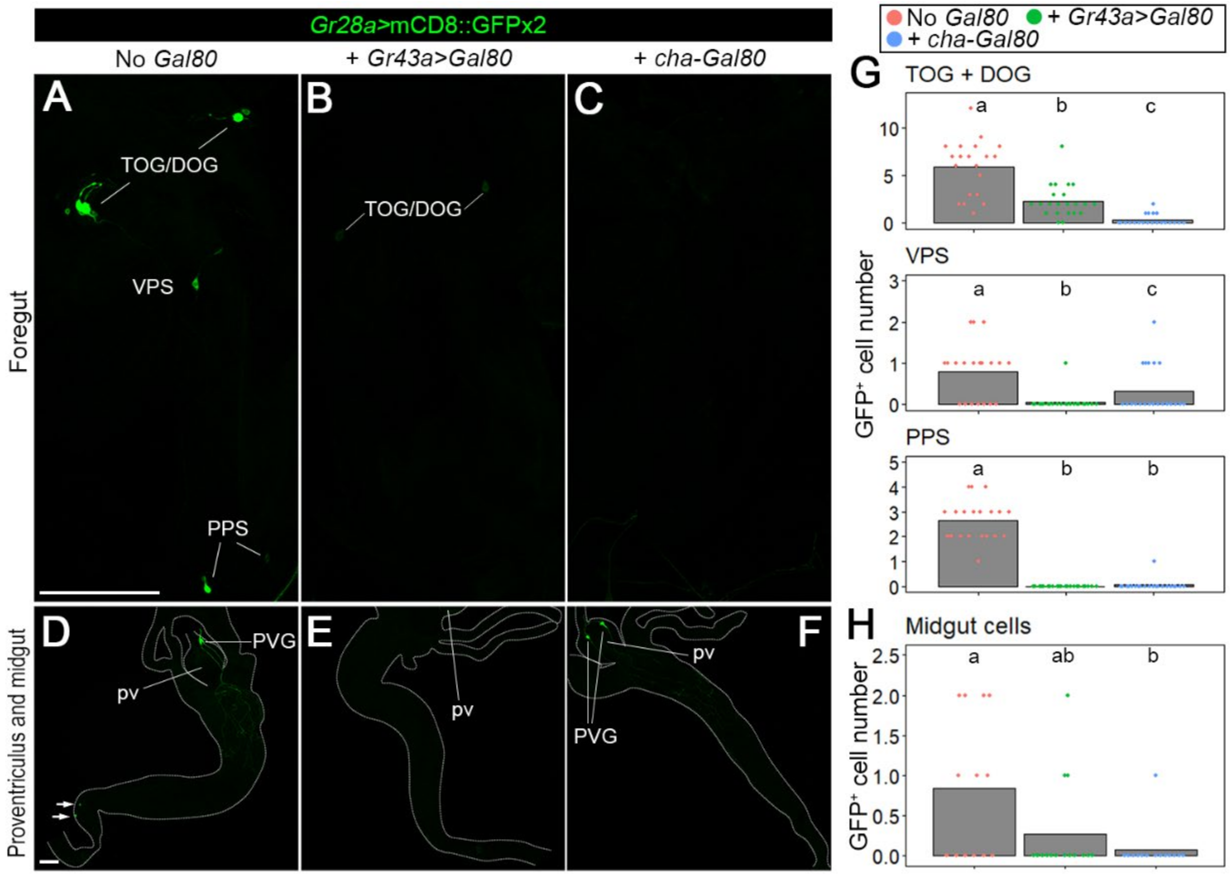

Fig. S5. ***Gr28a-Gal4*** **expression patterns in external and pharyngeal sensory neurons.**

(A–F) Projections of confocal stacks of the foregut with external (TOG/DOG) and pharyngeal (VPS and PPS) gustatory organs (A–C) and the proventriculus and midgut (D–F). *Gr28a-Gal4* activity visualized by two copies of *UAS-mCD8::GFP* in late 2nd instar larvae (A and D) was suppressed either by *Gr43a-LexA* driving *LexAop-Gal80* (B and E) or *cha-Gal80* (C and F). Arrows indicate GFP-positive midgut cells. Outlines of the intestine including the proventriculus (pv) and midgut are indicated by dotted lines. Scale bars = 100 μm. **(G and H)** Numbers of *Gr28a-Gal4*-positive cells in the sensory organs (TOG/DOG, VPS, and PPS) (G) and the midgut (H). Average values are shown in jitter plots. Sample sizes are 20–22 (G) and 13–15 (H) for each genotype. Different lowercase letters indicate statistically significant differences ($P < 0.05$; Steel-Dwass test).

Development • Supplementary information

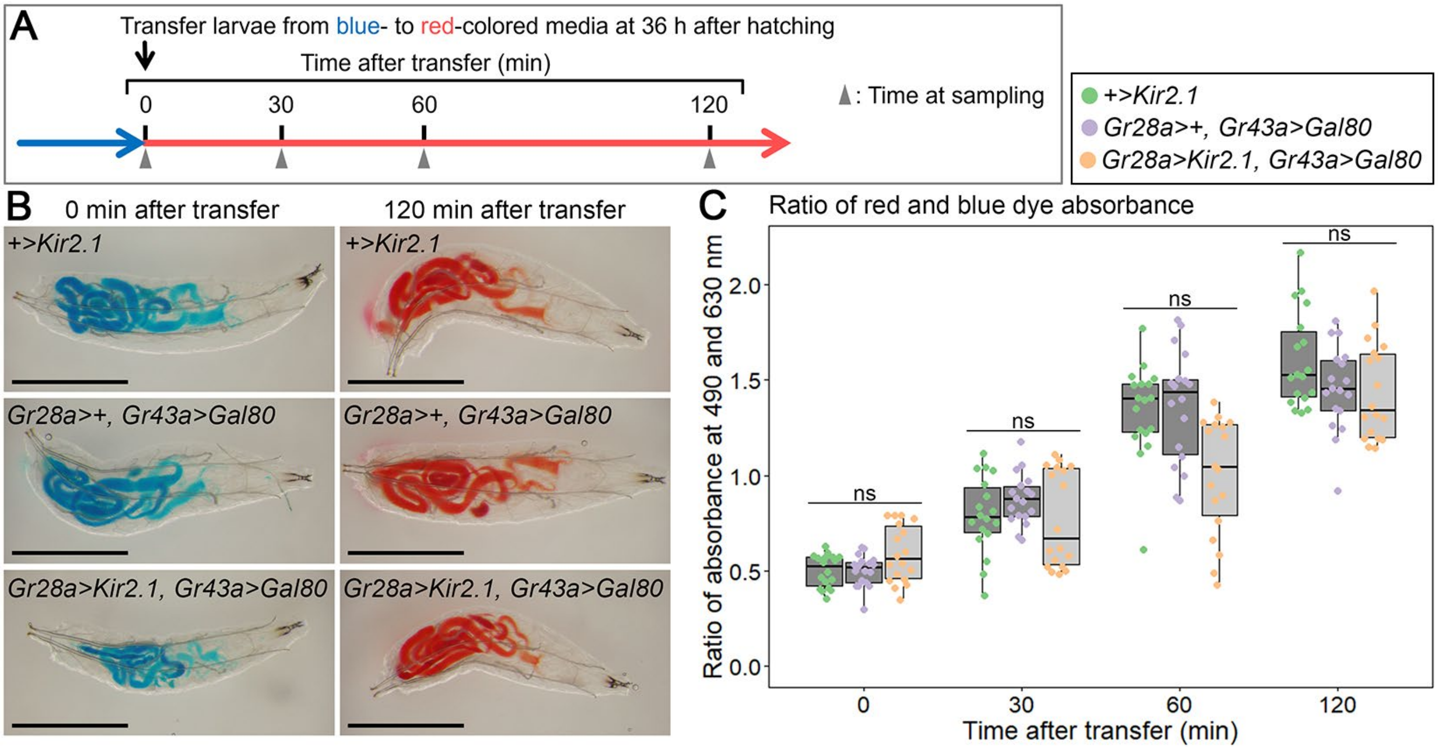

Fig. S6. Feeding behavior of *Gr28a*-expressing v'td neuron-silenced animals.

(A) Schematic diagram of the feeding behavior assay. Larvae were cultured on a blue-colored diet until 36 hAH, and then transferred to a red-colored diet. Larvae were sampled at indicated time points (arrowheads). **(B)** Images of control (*+>Kir2.1* and *Gr28a>+, Gr43a>Gal80*) and *Gr28a*-expressing v'td neuron-silenced larvae (*Gr28a>Kir2.1, Gr43a>Gal80*) reared on a blue-colored diet until 36 hAH (left panels) and those cultured on a red-colored for 120 min thereafter (right panels). Scale bars = 1 mm. **(C)** The ratio of absorbance at 490 nm (red) and 630 nm (blue) in the extracts of control and *Gr28a*-expressing v'td neuron-silenced larvae transferred from blue- to red-colored media at 36 hAH. Sample sizes are 10 in all groups (2 larvae were pooled for each sample at 36 hAH). Statistical analyses using Steel-Dwass test were performed for all groups in each experiment. ns, not significant ($P > 0.05$). The full result of the statistical analysis is shown in Table S4.

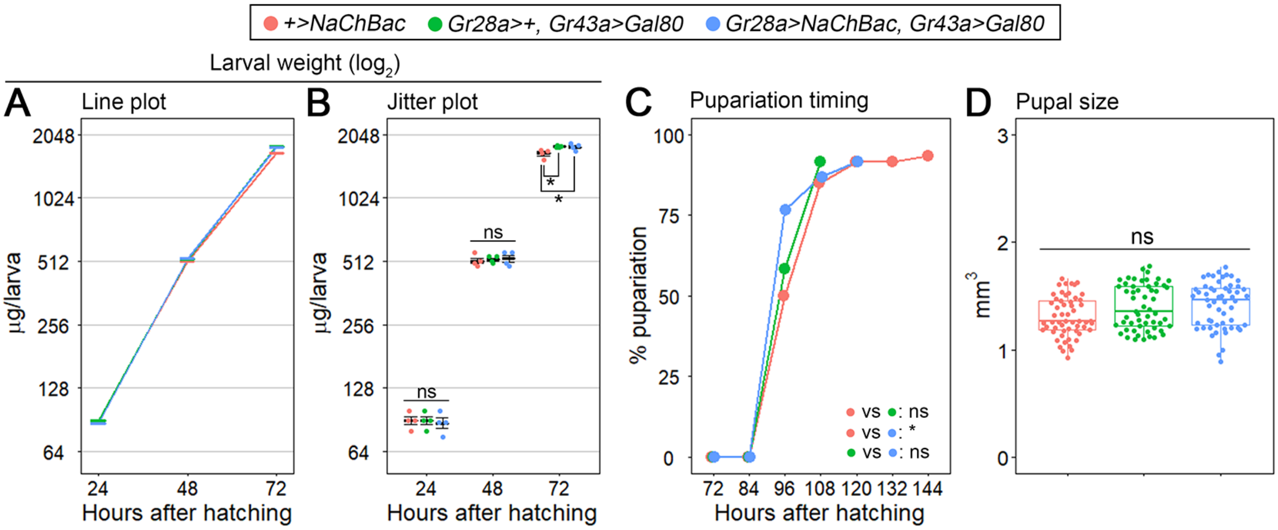

Fig. S7. Developmental phenotypes of *Gr28a*-expressing v'td neuron-activated animals.

(A and B) Body weight of control (+>*NaChBac* and *Gr28a*>+, *Gr43a*>*Gal80*) and *Gr28a*-expressing v'td neuron-activated larvae (*Gr28a*>*NaChBac*, *Gr43a*>*Gal80*) at indicted time points. In A, line plots with average values (bold lines) are shown for each genotype. In B, average values (bold black lines) with SE are shown in jitter plots. Sample sizes are 4 in all groups (5–10 larvae were pooled and body weight per larva was calculated for each sample). **(C)** Percentages of pupariated animals of each genotype at indicated time points. Sample sizes are 60 for each genotype. Results of statistical tests using the log-rank test are shown in the lower right (*$P < 0.05$). ns, not significant ($P > 0.05$). Raw *P*-values were multiplied by the number of independent tests (m = 3) in accordance with the Bonferroni correction method (Jafari and Ansari-Pour, 2019). Raw and corrected *P*-values are summarized in Table S4. **(D)** Pupal sizes of each genotype as shown in jitter and box plots. Sample sizes are 55 or 56 for each genotype. ns, not significant ($P > 0.05$; Steel-Dwass test).

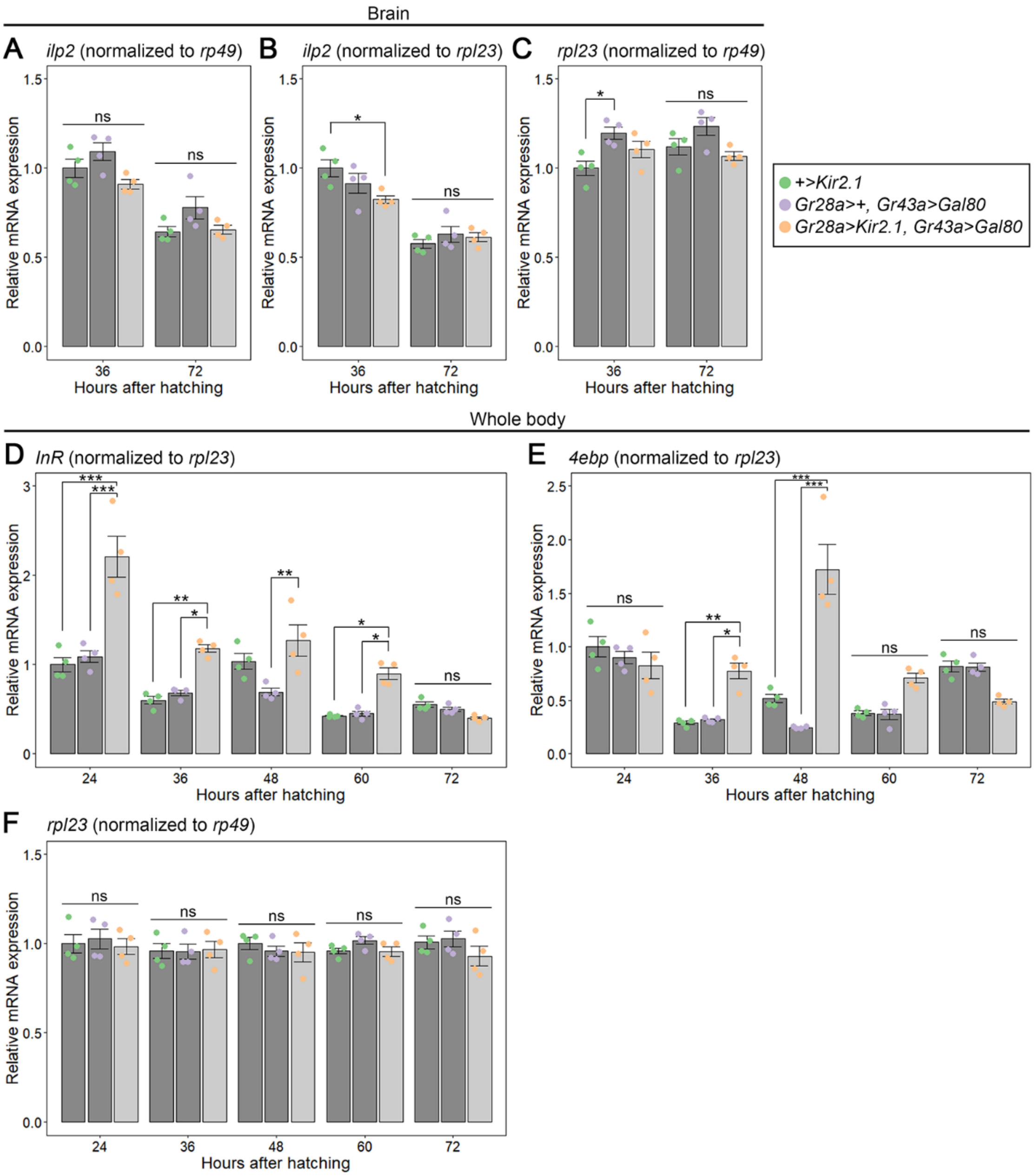

Fig. S8. Expression levels of *ilp2*, *InR*, and *4ebp* in *Gr28a*-expressing v'td neuron-silenced animals.

(A–C) Relative expression levels of *ilp2* (A and B) and *rpl23* (C) in the brains of control (*+>Kir2.1* and *Gr28a>+, Gr43a>Gal80*) and *Gr28a*-expressing v'td neuron-silenced larvae (*Gr28a>Kir2.1, Gr43a>Gal80*) at 36 and 72 hAH. Expression levels of *ilp2* were normalized to *rp49* (A) or *rpl23* (B). In C, *rpl23* expression levels were normalized to *rp49* to confirm constant expression of these reference genes. Average values of quadruplicated data sets are shown as bar graphs with SE and jitter plots. Statistical analyses using Tukey's test were performed for all groups. Asterisks show statistically significant differences (**P* < 0.05). ns, not significant (*P* > 0.05). The full result of the statistical analysis is shown in Table S4. **(D–F)** Relative expression levels of *InR* (D), *4ebp* (E), and *rpl23* (F) in control and *Gr28a*-expressing v'td neuron-silenced larvae at indicated time points. Expression levels of *InR* and *4ebp* were normalized to *rpl23* expression levels (D and E), and *rpl23* expression levels were normalized to *rp49* to further confirm its constant expression (F). Average values of quadruplicated data sets are shown as bar graphs with SE and jitter plots. Statistical analyses using Tukey's test were performed for all groups. Asterisks show statistically significant differences (**P* < 0.05; ***P* < 0.01; ****P* < 0.001). ns, not significant (*P* > 0.05). The full result of the statistical analysis is shown in Table S4.

Table S1. GRN developmental timing screen results. BDSC, Bloomington *Drosophila* Stock Center.

Click here to download Table S1

Table S2. Fly stocks used in this study.

Genotype	Chromosomes	Source	Stock ID
w^{1118}	1	BDSC	5905
w^{1118} (an injection strain for $Gr28a^{GAL4}$)	1	WellGenetics	-
w^{1118}; $Gr28a^{GAL4}$	1;2	This study	-
w^{1118}; *ilp2-Gal4*	1;2	BDSC	37516
w^{1118};; *R73B01-Gal4*	1;3	BDSC	39809
w^{1118};; *R22C07-Gal4*	1;3	BDSC	48975
w^{*}; *UAS-Kir2.1*	1;2	BDCS	6596
$y^{1}w^{*}$; *UAS-NaChBac*	1;2	BDSC	9466
$y^{1}w^{*}$; *UAS-mCD8::GFP x2*	1;2	BDCS	5137
w^{*}; *UAS-2xEGFP*	1;2	BDCS	6874
w^{1118}; L^{1}*/CyO; UAS-Syt::GFP, UAS-Denmark*	1;2;3	BDSC	33065
w^{*}; *LexAop-mCherry-HA/CyO; UAS-mCD8::GFP*	1;2;3	Yamanaka lab	-
w^{*}; *LexAop-spGFP*$_{11}$*::CD4; UAS-spGFP*$_{1\text{-}10}$*::Nrx/TM6B*	1;2;3	Yamanaka lab	-
w; ilp2-LexA; Dr/Tb*	1;2;3	Gong lab	-
w^{*}; *Gr43a-LexA*	1;3	Amrein lab	-
w^{*}; *LexAop-Gal80*	1;2	BDSC	32214
w^{*};; *cha-Gal80*	1;3	Kitamoto lab	-

BDSC, Bloomington *Drosophila* Stock Center.

Table S3. Genotypes of flies used in this study.

Abbreviations of fly genotypes	Genotypes of parents	
+>Kir2.1	w^{*}; UAS-Kir2.1	w^{1118}
Gr>Kir2.1	w^{*}; UAS-Kir2.1	w^{*}; Gr-Gal4 (on 2nd or 3rd chr)
Gr28a>+	w^{1118}	w^{*}; Gr28a-Gal4
Gr28a>Kir2.1	w^{*}; UAS-Kir2.1	w^{*}; Gr28a-Gal4
Gr28b.c>+	w^{1118}	w^{*};; Gr28b.c-Gal4
Gr28b.c>Kir2.1	w^{*}; UAS-Kir2.1	w^{*};; Gr28b.c-Gal4
Gr89a>+	w^{1118}	w^{*}; Gr89a-Gal4/CyO-GFP
Gr89a>Kir2.1	w^{*}; UAS-Kir2.1	w^{*}; Gr89a-Gal4/CyO-GFP
R73B01>+	w^{1118}	w^{1118};; R73B01-Gal4
R73B01>Kir2.1	w^{*}; UAS-Kir2.1	w^{1118};; R73B01-Gal4
R22C07>+	w^{1118}	w^{1118};; R22C07-Gal4
R22C07>Kir2.1	w^{*}; UAS-Kir2.1	w^{1118};; R22C07-Gal4
+>Kir2.1 x2	w^{*}; UAS-Kir2.1	w^{*}; UAS-Kir2.1
Gr28b.c x2>+	w^{*};; Gr28b.c-Gal4	w^{*};; Gr28b.c-Gal4
Gr28b.c x2>Kir2.1 x2	w^{*}; UAS-Kir2.1; Gr28b.c-Gal4	w^{*}; UAS-Kir2.1; Gr28b.c-Gal4
Gr89a>Kir2.1 x2	w^{*}; Gr89a-Gal4, UAS-Kir2.1/CyO-GFP	w^{*}; UAS-Kir2.1
Gr28a>+, Gr43a>Gal80	w^{1118}	w^{*}; Gr28a-Gal4, LexAop-Gal80; Gr43a-LexA
Gr28a>Kir2.1, Gr43a>Gal80	w^{*}; UAS-Kir2.1	w^{*}; Gr28a-Gal4, LexAop-Gal80; Gr43a-LexA
Gr28a>+, cha-Gal80	w^{1118}	w^{*}; Gr28a-Gal4; cha-Gal80
Gr28a>Kir2.1, cha-Gal80	w^{*}; UAS-Kir2.1	w^{*}; Gr28a-Gal4; cha-Gal80
+>NaChBac	$y^{1}w^{*}$; UAS-NaChBac	w^{1118}
Gr28a>NaChBac, Gr43a>Gal80	$y^{1}w^{*}$; UAS-NaChBac	w^{*}; Gr28a-Gal4, LexAop-Gal80; Gr43a-LexA
+/+	w^{1118} *	w^{1118} *
$Gr28a^{GAL4}$/+	w^{1118} *	w^{1118}; $Gr28a^{GAL4}$
$Gr28a^{GAL4}$/$Gr28a^{GAL4}$	w^{1118}; $Gr28a^{GAL4}$	w^{1118}; $Gr28a^{GAL4}$
Gr28a>mCD8::GFP x2	$y^{1}w^{*}$; UAS-mCD8::GFP x2	w^{*}; Gr28a-Gal4
Gr28b.c>mCD8::GFP x2	$y^{1}w^{*}$; UAS-mCD8::GFP x2	w^{*};; Gr28b.c-Gal4
Gr89a>mCD8::GFP x2	$y^{1}w^{*}$; UAS-mCD8::GFP x2	w^{*}; Gr89a-Gal4/CyO-GFP
R73B01>mCD8::GFP x2	$y^{1}w^{*}$; UAS-mCD8::GFP x2	w^{1118};; R73B01-Gal4
R22C07>mCD8::GFP x2	$y^{1}w^{*}$; UAS-mCD8::GFP x2	w^{1118};; R22C07-Gal4
Gr28a>mCD8::GFP x2, Gr43>Gal80	$y^{1}w^{*}$; UAS-mCD8::GFP x2	w^{*}; Gr28a-Gal4, LexAop-Gal80; Gr43a-LexA
Gr28a>mCD8::GFP x2, cha-Gal80	$y^{1}w^{*}$; UAS-mCD8::GFP x2	w^{*}; Gr28a-Gal4; cha-Gal80
$Gr28a^{GAL4}$>mCD8::GFP x2	$y^{1}w^{*}$; UAS-mCD8::GFP x2	w^{1118}; $Gr28a^{GAL4}$
Gr39a.b>2xEGFP	w^{*}; UAS-2xEGFP	w^{*}; Sp/CyO; Gr39a.b-Gal4
ilp2>Syt::GFP Denmark	w^{1118}; L^{1}/CyO; UAS-Syt::GFP, UAS-Denmark	w^{1118}; ilp2-Gal4
Gr28a>mCD8::GFP, ilp2>mCherry	w^{*}; LexAop-mCherry-HA/CyO; UAS-mCD8::GFP	w^{*}; ilp2-LexA; Gr28a-Gal4
Gr28a-Gal4>UAS-$spGFP_{1-10}$::Nrx, +>LexAop-spGFP11::CD4	w^{*}; LexAop-$spGFP_{11}$::CD4; UAS-$spGFP_{1-10}$::Nrx/TM6B	w^{*}; Sp/CyO; Gr28a-Gal4
+>UAS-$spGFP_{1-10}$::Nrx, ilp2-LexA>LexAop-spGFP11::CD4	w^{*}; LexAop-$spGFP_{11}$::CD4; UAS-$spGFP_{1-10}$::Nrx/TM6B	w^{*}; ilp2-LexA; Dr/Tb
Gr28a-Gal4>UAS-$spGFP_{1-10}$::Nrx, ilp2-LexA>LexAop-spGFP11::CD4	w^{*}; LexAop-$spGFP_{11}$::CD4; UAS-$spGFP_{1-10}$::Nrx/TM6B	w^{*}; ilp2-LexA; Gr28a-Gal4

*an injection strain for *Gr28a* mutagenesis

Development • Supplementary information

Table S4. Exact, raw, and corrected *P*-values.

Red values indicate corrected *P*-values under 0.05. For Fig. 4I and S2K, statistical analyses were performed using the log-rank test. Raw *P*-values under 0.05 were multiplied by the number of independent tests (m = 21 and 42, respectively) in accordance with the Bonferroni correction method (Jafari and Ansari-Pour, 2019). For the other data, colored background indicates results of statistical analyses at the same time points. *, Steel-Dwass test. **, Tukey's test.

Click here to download Table S4
