# Supplementary material for: Internal sensory neurons regulate stage-specific growth in Drosophila
Source: Development. 2022 Oct 28;149(21):dev200440. doi: 10.1242/dev.200440 (PMC10496149; doi:10.1242/dev.200440)
Supplement: Supplementary file 1 [file develop-149-200440_review_history.pdf]

Internal sensory neurons regulate stage-specific growth in *Drosophila*

Yuya Ohhara and Naoki Yamanaka
DOI: 10.1242/dev.200440

Editor: Irene Miguel-Aliaga

Review timeline

Original submission:	6 December 2021
Editorial decision:	11 January 2022
First revision received:	21 April 2022
Editorial decision:	17 May 2022
Second revision received:	31 August 2022
Accepted:	22 September 2022

Original submission

First decision letter

MS ID#: DEVELOP/2021/200440

MS TITLE: Internal sensory neurons innervate insulin-producing cells to regulate stage-specific growth in Drosophila

AUTHORS: Yuya Ohhara and Naoki Yamanaka

I have now received all the referees' reports on the above manuscript, and have reached a decision. The referees' comments are appended below, or you can access them online: please go to BenchPress and click on the 'Manuscripts with Decisions' queue in the Author Area.

As you will see, the referees express considerable interest in your work, but have some significant criticisms and recommend a substantial revision of your manuscript before we can consider publication. Having gone through the reviews (and this being a topic close to my expertise), I do agree that all their concerns need to be addressed. If you are able to revise the manuscript along the lines suggested, I will be happy to receive a revised version of the manuscript. Your revised paper will be re-reviewed by one or more of the original referees, and acceptance of your manuscript will depend on your addressing satisfactorily the reviewers' major concerns. Please also note that Development will normally permit only one round of major revision.

We are aware that you may be experiencing disruption to the normal running of your lab that make experimental revisions challenging. If it would be helpful, we encourage you to contact us to discuss your revision in greater detail. Please send us a point-by-point response indicating where you are able to address concerns raised (either experimentally or by changes to the text) and where you will not be able to do so within the normal timeframe of a revision. We will then provide further guidance. Please also note that we are happy to extend revision timeframes as necessary.

Please attend to all of the reviewers' comments and ensure that you clearly highlight all changes made in the revised manuscript. Please avoid using 'Tracked changes' in Word files as these are lost in PDF conversion. I should be grateful if you would also provide a point-by-point response detailing how you have dealt with the points raised by the reviewers in the 'Response to Reviewers' box. If you do not agree with any of their criticisms or suggestions please explain clearly why this is so.

© 2022. Published by The Company of Biologists under the terms of the Creative Commons Attribution License (https://creativecommons.org/licenses/by/4.0/).

Reviewer 1

Advance summary and potential significance to field

This study investigates the role of chemosensory inputs on growth and development. To identify gustatory receptor neurons that couple the chemosensory system with larval development, the authors employed 66 different Gr-Gal4 lines, whose regulatory elements are derived from corresponding Gr genes, to silence neuronal activity and screened for altered timing of pupariation. Their screen identify Gr28a-Gal4 positive neurons as required for normal larval growth and developmental timing. They go on to characterize these Gr28a-expressing neurons and identify different neuronal populations. Using Gal80 to restrict expression to different subpopulations of neurons and show that the neurons controlling developmental timing correspond to peripheral body wall sensory neurons that project onto the insulin-producing cells. They next show that silencing of the Gr28a-expressing neurons blocks Dilp release from the IPCs and suppress larval growth specifically during mid-larval period.

Numerous studies have shown a link between internal nutrient-sensing mechanisms and Dilp-mediated larval growth. This study provides an interesting novel link between the chemosensory system and larval growth. This reviewer therefore recommends the manuscript for publication in Development with some revision (see below).

Comments for the author

Major points:

- It would be nice to see if a Gr28a mutant animals can replicate the phenotypes observed upon Gr28a-Gal4 RNAi mediated knockdown

- The authors do not show that the Gr28a-positive neurons (v'td) interacts physically with the IPCs. This could be done using GRASP.

- The authors show that silencing Gr28a-positive neurons affect growth in the mid larval stage and that this corresponds to retention of Dilps in the IPCs and a difference 4EBP expression. The authors should show that the expression of brain dilps are not affected (increased) in this condition. Furthermore, they should include an additional IIS target to show that it behaves similarly to 4EBP, e.g. the InR. Finally, they could look at circulating levels of Dilp2 at the 2nd larval stage using Elisa.

- Silencing of Gr28a-positive neurons slows down growth at the mid larval stage. The authors should address how activation of the same neurons affect: 1) insulin secretion, 2) growth, and 3) pupal size?

- How does silencing of Gr28a-positive neurons affect feeding at different stages of development? This is an important point as it is conceivable that the chemosensory system could affect feeding behavior.

Reviewer 2

Advance summary and potential significance to field

In this paper, Ohhara and Yamanaka, identify a group of sensory neurons important for controlling systemic insulin signaling and body growth in Drosophila larvae.

The authors began by postulating that since gustatory receptor neurons (GRNs) play an important role in sensing environmental and nutrient cues, that they may regulate growth. They use a panel of GRN-gal4 lines to silence specific GRNs and identify the Gr28a neurons as being important for normal body growth and development. They further pinpoint a subset of Gr28a body wall neurons as being the key regulators of body growth. And they present evidence to suggest that this regulation is mediated by direct connection between the Gr28a neurons and the brain insulin-

© 2022. Published by The Company of Biologists under the terms of the Creative Commons Attribution License (https://creativecommons.org/licenses/by/4.0/).

producing cells, and consequent stimulation of insulin release and activation of systemic insulin signaling.

Overall, this is a very nice study that provides new insights into the neuronal control of insulin signaling and growth. I think it will be of interest to those interested in growth control and in Drosophila physiology and development.

Comments for the author

Overall this is a solid paper. It doesn't pinpoint exactly what the Gr28a neurons are sensing or responding to to control insulin signaling, and it doesn't show exactly how the Gr28a neurons modulate the IPCs, but it is still a fascinating and important discovery that I would encourage for publication after revision. I have a few things for the authors to consider in their revision. Most of my major comments concern the link between the Gr28a neurons and insulin signaling.

1, In Fig 5A, B the authors nicely show that silencing of the Gr28a neurons leads to reduced larval size compared to age-matched controls. However, in 5C, then use calculations of the relative change in body size between different times (24, 48 72hrs) to suggest that following silencing of the Gr28 neurons, “growth is predominantly downregulated from 24 to 48 291 hAH in the v’td-silenced animals but it is restored thereafter”. However, I wonder whether the interpretation is that straightforward. It certainly is the case that the relative growth increase from 48 to 72h is similar between control and Gr28a silenced animals, but the absolute growth (mass) increase is still much higher in the controls, so I’m not sure that the idea of a temporary growth suppression is so clear cut. This is important because the authors go on to interpret their insulin signaling data (Fig 5E-G) in the context of this selective growth effect at 48hrs.

2, Fig 5E-G explores the effect of Gr28a silencing on insulin signaling. The authors use two readouts: dILP2 IPC levels (as an indirect measure of release/retention) and 4ebp mRNA levels (as a readout of FOXO function). While both do provide a measure of insulin signaling, they are somewhat indirect and would be bolstered by additional approaches. I would suggest a) trying to measure hemolymph dilp2 levels (using the ELISA method with the HA-tagged version of dilp2 developed by the Kim lab – Park et al PLOS Genet, 2014), although this could be tricky with young larvae; and/or b) looking at phospho-Akt by WB or immunostaining. This is a pretty straightforward and widely used approach to assay for alterations in insulin signaling.

3, The authors interpret their data in Fig 5E-G as suggesting that insulin signaling is temporarily suppressed in a time window at ~48hAH. In Fig 5F, it is true that the dilp2 levels are particularly higher in the silence neurons at 48-60h, they are still significantly higher at all time points after 24h suggesting a persistent suppression of insulin signaling. Also, in Fig 5G, there is a significant increase in 4ebp only at 48h, which the authors use as further evidence of the temporary suppression of insulin signaling. However, at the 72h point, 4ebp levels are lower in the Gr28a silenced neurons, which could be interpreted as an increase in insulin signal. One issue with the expts is the RpL32 mRNA levels are used to control/correct for changes in 4ebp mRNA level. This is potentially problematic since RpL32 mRNA levels do change quite a lot at each stage of larval development (e.g., Flybase modENCODE dev RNA seq data) and they are altered by insulin signaling. It might be worthwhile for the authors to try a few other control mRNAs and see if the selective changes in 4ebp mRNA levels at different timepoints hold true. In addition, the additional experiments I suggest in point #2 above (e.g phosphor-Akt measurements) should also help clarify the changes in insulin signaling at different time points.

Minor points

i, Fig 4D – it would be useful to include statistical analyses of these date (maybe log rank test). It looks like the Gr28a silenced neurons with cha-Gal80 still do show a small, but significant (~12hrs) delay in development, while the Gr43a-Gal80 larvae are delayed but not as strongly as the complete Gr28 silenced animals. These results would suggest some role for the non v’td neurons in controlling growth.
ii, I think the overall finding - GRNs controlling insulin and growth - is fascinating. One thing I wondered, is whether it is signaling through the Gr28a receptor itself that is important for growth. Is there any evidence for Gr28 receptors themselves being important for larval development?

© 2022. Published by The Company of Biologists under the terms of the Creative Commons Attribution License (https://creativecommons.org/licenses/by/4.0/).

Reviewer 3

Advance summary and potential significance to field

Using a screening approach, the authors show that Gr28a-Gal4 expressing neurons seem to be specifically involved in developmental progression from the larval to the pupal stage. The authors use genetic means to narrow these down to a subset of six Gr28a-Gal4-positive v'td(2) neurons, which might act as internal sensors. The authors' data indicate that Gr28a cells directly regulate Insulin release from IPCs as silencing of Gr28a-Gal4-positive cells increased Ilp2 levels in IPCs and transiently increased 4EBP mRNA levels. The authors propose that v'td neurons transiently regulate Insulin release to promote developmental progression.

Comments for the author

Based on the authors' data and as outlined below, the involvement of G28a-positive v'td neurons is not convincing, as in my view it cannot be excluded that expression in other cells might induce the observed phenotype. Moreover, direct connectivity between v'td neurons and IPCs has not been demonstrated by the authors, although this is stated in the title. Overall, the paper is quite descriptive and does not provide compelling evidence for a novel developmental mechanisms regulating larval to pupal transition. Specific point are:

1. While I do not doubt the screening results per se, I am puzzled by the fact that other lines expressing in the described v'td neurons did not show the same developmental delay with Kir2.1 silencing, e.g. Gr89a-Gal4 and Gr28b.c-Gal4 (see Qian et al., J Neurosci 2019) also express in these cells yet do not show the same developmental delay (see Fig.1). The authors observe a transient developmental effect of Gr28a-v'td neuron silencing between 24-72 haH. Late 2nd instar larvae (h after hatching?) were used for expression analysis, while the data indicate that growth retardation upon Gr28a-Gal4 silencing is starting much earlier, possibly even before 24h after hatching. As Gal4 expression patterns can change over time, this suggests that either there is expression of Gr28a-Gal4 in other tissues/neurons that is not blocked by the authors' Gal80 approach (transiently or permanently), or that the other Gal4 lines do not express during the developmental window proposed by the authors to be the main driver of the effect. The whole paper critically relies on the used Gr28a-Gal4 line, which raises some doubts about the specific link to the proposed v'td neurons. Addressing this would require very detailed description of the developmental expression of this line in neuronal and non-neuronal tissues and showing why other v'td neuron-expressing lines do not display the same effect.

2. Along the same lines as above, specificity of the authors' findings should be shown using additional independent lines expressing in the described neuronal subset, e.g. 22C07-Gal4 or other lines characterized in Qian et al., J Neurosci 2019.

3. There is an elaborate description of the involvement of Gr`s in nutrient sensing in the introduction and discussion, thus one would expect that the authors had checked for the involvement of Gr28a v'td neurons in sensing nutrients externally or internally. The authors suggest that Gr28a-expressing v'td cells are involved in RNA sensing as suggested for Gr28a in previous work (Mishra et al., PLOS Biol 2018). However, this was not tested and the conclusion that Gr28a-Gal4 labelled v'td neurons are gustatory receptor neurons regulating ILP production is somewhat overstated. V'td neurons have so far been implicated in sensing CO2 and noxious light (Hückesfeld et al. et al. Elife 2021, Imambocus et al., Curr Biol 2021) yet internal sensory functions as proposed here are not shown by the authors. The authors should keep in mind that Gal4 lines based on enhancer fragments as used in this study do not necessarily represent the expression of the respective Gr, including Gr28a. In several places in the manuscript, this distinction is not so clear and the authors discuss a lot about the role of Gr28a without testing its function, which could have been performed e.g with Gr28a-RNAi.

4. The title is overstating the findings ("Internal sensory neurons innervate insulin-producing cells…"), as direct connectivity of Gr28a-Gal4-expressing neurons with IPCs has not been convincingly demonstrated. Close proximity of respective axonal and dendritic arbors is not a sufficient criterion for connectivity. The SEZ is densely packed and many of the neurons with projections in this region are not necessarily directly connected. At the minimum, a synaptic GRASP

© 2022. Published by The Company of Biologists under the terms of the Creative Commons Attribution License (https://creativecommons.org/licenses/by/4.0/).

experiment would be necessary to indicate direct connectivity. The authors could have easily performed this, as these tools are commonly available and standard in the field. Testing functional connectivity, even if indirect, would be an even better proof of a causal link between v'td neurons and IPCs, e.g. testing if activation (e.g. optogenetically) of the identified v'td neurons increases IPC activity.

5. Body weight seems to be already affected at 24 haH by silencing of Gr28b-Gal4 expressing cells (Fig. 5A). However, statistical analysis for Fig. 5A-D was not performed. This makes it difficult to evaluate the data and effects. The authors claim that the effect at 24h hAH is small, yet at the scale of the figure it is impossible to judge that. Body weight might well be similarly reduced as at later stages. Moreover, Fig. 5C and D are confusing and not well explained in the legend. It looks like weight and surface was simply normalized to 1 at 24h, which does not add any information.

6. It is not clear to me how Ilp2 levels were quantified. Was there any normalization performed to correct for overall sample differences? The methods lack sufficient detail to reconcile how this was performed.

7. No causal link between Ilp2 accumulation in IPCs and the observed transient developmental delay upon v'td neuron silencing has been provided. Can the developmental delay be rescued by increasing IPC activity or Ilp2 release?

Minor points:

1. Fig 2K`and K``: Gr28a-Gal4 is expressed in v'td and HCG/PVG neurons projecting to the SEZ from the posterior and anterior end, respectively. The axonal bundle from HCG/PVG neurons in the SEZ in Fig. 2K' appears thicker than shown later in Fig. 3C. Are axons from additional (gustatory) Gr28a expressing neurons present as well?

2. Statistical analysis is only provided for Fig. 5F and G, but not Fig. 5A to D. Moreover, while a supplementary table is appropriate to show all P values, it is very inconvenient to not indicate the relevant P values in the figure. This makes an assessment of the data unnecessarily complicated.

First revision

Author response to reviewers' comments

Below are the specific, point-by-point responses to the reviewers' comments:

Reviewer 1:

1. It would be nice to see if a Gr28a mutant animals can replicate the phenotypes observed upon Gr28a- Gal4 RNAi mediated knockdown.

We generated a null mutant of *Gr28a* by replacing the whole gene with a Gal4-containing cassette (Fig. S3). Homozygous *Gr28a*^{GAL4} mutants showed growth rate reduction and pupariation delay similar to *Gr28a*-expressing v'td neuron-silenced larvae, but the phenotype was much milder (Fig. 4, 5, and S3). This suggests that *Gr28a*-expressing v'td neuron activity is not completely suppressed in *Gr28a* mutants. We also discussed these results in Discussion (p. 16, lines 330-350).

2. The authors do not show that the Gr28a-positive neurons (v'td) interacts physically with the IPCs. This could be done using GRASP.

We performed GRASP analysis and the results are now shown in Fig 3E-G''. Reconstituted GFP signals were clearly observed in the SEZ between neurons labeled by *Gr28a-Gal4* and *ilp2-LexA*, indicating physical interactions between *Gr28a*-expressing neurons and IPCs.

© 2022. Published by The Company of Biologists under the terms of the Creative Commons Attribution License (https://creativecommons.org/licenses/by/4.0/).

3. The authors show that silencing Gr28a-positive neurons affect growth in the mid larval stage and that this corresponds to retention of Dilps in the IPCs and a difference 4EBP expression. The authors should show that the expression of brain dilps are not affected (increased) in this condition. Furthermore, they should include an additional IIS target to show that it behaves similarly to 4EBP, e.g. the InR. Finally, they could look at circulating levels of Dilp2 at the 2nd larval stage using Elisa.

We performed qRT-PCR and confirmed that *ilp2* expression is not upregulated in *Gr28a*-expressing v'td neuron-silenced larvae (Fig. S8A-C). We also quantified *InR* expression as suggested and obtained results similar to *4ebp* (Fig. S8D).
As for the measurement of the levels of circulating ILP2, we found that it is technically too challenging due to the small body size of 2nd instar larvae. We therefore instead quantified the levels of phospho-Akt in peripheral tissues as a readout of insulin signaling as suggested by Reviewer 2. The results (Fig. 5I-K) further indicate that insulin signaling is temporarily suppressed in the v'td-silenced animals during the mid-larval period.

4. Silencing of Gr28a-positive neurons slows down growth at the mid larval stage. The authors should address how activation of the same neurons affect: 1) insulin secretion, 2) growth, and 3) pupal size?

We performed continuous activation of *Gr28a*-expressing v'td neurons by NaChBac expression, which caused neither acceleration of larval growth nor an increase in pupal volume (Fig. S7). These results suggest that the v'td neuron activity is not sufficient for promoting larval growth.

5. How does silencing of Gr28a-positive neurons affect feeding at different stages of development? This is an important point as it is conceivable that the chemosensory system could affect feeding behavior.

We performed a feeding assay as shown in Fig. S6. The result revealed that feeding is not significantly altered in the v'td-silenced larvae (Fig. S6C), suggesting that growth rate suppression is not caused by feeding defects in these animals.

We thank this reviewer for all their important suggestions, which greatly helped us improve our manuscript.

Reviewer 2:

Overall this is a solid paper. It doesn't pinpoint exactly what the Gr28a neurons are sensing or responding to to control insulin signaling, and it doesn't show exactly how the Gr28a neurons modulate the IPCs, but it is still a fascinating and important discovery that I would encourage for publication after revision. I have a few things for the authors to consider in their revision. Most of my major comments concern the link between the Gr28a neurons and insulin signaling.

We thank the reviewer for their encouraging comments.

1. In Fig 5A, B the authors nicely show that silencing of the Gr28a neurons leads to reduced larval size compared to age-matched controls. However, in 5C, then use calculations of the relative change in body size between different times (24, 48, 72hrs) to suggest that following silencing of the Gr28 neurons, "growth is predominantly downregulated from 24 to 48 hAH in the v'td-silenced animals, but it is restored thereafter". However, I wonder whether the interpretation is that straightforward. It certainly is the case that the relative growth increase from 48 to 72h is similar between control and Gr28a silenced animals, but the absolute growth (mass) increase is still much higher in the controls, so I'm not sure that the idea of a temporary growth suppression is so clear cut. This is important because the authors go on to interpret their insulin signaling data (Fig 5E-G) in the context of this selective growth effect at 48hrs.

We agree with the reviewer that our original explanation about the phenotype was premature. In the revised manuscript, we avoided the statement that the growth was

© 2022. Published by The Company of Biologists under the terms of the Creative Commons Attribution License (https://creativecommons.org/licenses/by/4.0/).

“restored” after 48 hAH, and we now simply focus on describing growth defects observed during the mid-larval stage (Fig. 5). In Fig. 5A-D, we also separated line plots and jitter plots, so the readers can interpret the growth defect phenotype observed in *Gr28a*-expressing v’td neuron-silenced larvae more directly by themselves.

2. Fig 5E-G explores the effect of Gr28a silencing on insulin signaling. The authors use two readouts: dILP2 IPC levels (as an indirect measure of release/retention) and 4ebp mRNA levels (as a readout of FOXO function). While both do provide a measure of insulin signaling, they are somewhat indirect and would be bolstered by additional approaches. I would suggest a) trying to measure hemolymph dilp2 levels (using the ELISA method with the HA-tagged version of dilp2 developed by the Kim lab – Park et al PLOS Genet, 2014), although this could be tricky with young larvae; and/or b) looking at phospho- Akt by WB or immunostaining. This is a pretty straightforward and widely used approach to assay for alterations in insulin signaling.

We thank the reviewer for this important suggestion. As we explained in response to Reviewer #1 above, we found that the measurement of the levels of circulating ILP2 is technically too challenging due to the small body size of 2nd instar larvae. We therefore instead quantified the levels of phospho- Akt in peripheral tissues by immunostaining as suggested. The results (Fig. 5I-K) indicate that insulin signaling is temporarily suppressed in the v’td-silenced animals during the mid-larval period, consistent with the other results.

3. The authors interpret their data in Fig 5E-G as suggesting that insulin signaling is temporarily suppressed in a time window at ~48hAH. In Fig 5F, it is true that the dilp2 levels are particularly higher in the silence neurons at 48-60h, they are still significantly higher at all time points after 24h suggesting a persistent suppression of insulin signaling. Also, in Fig 5G, there is a significant increase in 4ebp only at 48h, which the authors use as further evidence of the temporary suppression of insulin signaling. However, at the 72h point, 4ebp levels are lower in the Gr28a silenced neurons, which could be interpreted as an increase in insulin signal. One issue with the expts is the RpL32 mRNA levels are used to control/correct for changes in 4ebp mRNA level. This is potentially problematic since RpL32 mRNA levels do change quite a lot at each stage of larval development (e.g., Flybase modENCODE dev RNA seq data) and they are altered by insulin signaling. It might be worthwhile for the authors to try a few other control mRNAs and see if the selective changes in 4ebp mRNA levels at different timepoints hold true. In addition, the additional experiments I suggest in point #2 above (e.g phosphor-Akt measurements) should also help clarify the changes in insulin signaling at different time points.

We selected *rpl23* as a second reference gene, as it has been previously reported that *rpl23*, as well as *rp49*, shows stable expression during larval development (Danielsen et al., 2014, *PLoS Genetics*).
Normalized data using this secondary reference gene, as well as the comparison of expression levels between the two reference genes, are now shown in Fig. S8. Combined with the p-Akt immunostaining mentioned above, these additional results further support the idea that insulin signaling is temporarily suppressed in the v’td-silenced animals during the mid-larval period.

Minor points

i. Fig 4D – it would be useful to include statistical analyses of these date (maybe log rank test). It looks like the Gr28a silenced neurons with cha-Gal80 still do show a small, but significant (~12hrs) delay in development, while the Gr43a-Gal80 larvae are delayed but not as strongly as the complete Gr28 silenced animals. These results would suggest some role for the non v’td neurons in controlling growth.

We performed the log-rank test for the data in Fig. 4I and confirmed that *Gr28a-Gal4*-mediated silencing with *cha-Gal80* indeed shows a significant developmental delay. The results are now explained on p. 10, lines 194-207.

ii. I think the overall finding - GRNs controlling insulin and growth - is fascinating. One thing I wondered, is whether it is signaling through the Gr28a receptor itself that is important for growth. Is there any evidence for Gr28 receptors themselves being important for larval development?

© 2022. Published by The Company of Biologists under the terms of the Creative Commons Attribution License (https://creativecommons.org/licenses/by/4.0/).

To answer this question, we generated a null mutant of *Gr28a* by replacing the whole gene with a Gal4-containing cassette (Fig. S3). Homozygous *Gr28a*^{GAL4} mutants showed growth rate reduction and pupariation delay similar to *Gr28a*-expressing v'td neuron-silenced larvae, but the phenotype was much milder (Fig. 4, 5, and S3). This suggests that *Gr28a*-expressing v'td neuron activity is not completely suppressed in *Gr28a* mutants. We also discussed these results in Discussion (p. 16, lines 330-350).

We thank the reviewer again for all their valuable suggestions.

Reviewer 3:

1. While I do not doubt the screening results per se, I am puzzled by the fact that other lines expressing in the described v'td neurons did not show the same developmental delay with Kir2.1 silencing, e.g. Gr89a-Gal4 and Gr28b.c-Gal4 (see Qian et al., J Neurosci 2019) also express in these cells yet do not show the same developmental delay (see Fig.1). The authors observe a transient developmental effect of Gr28a-v'td neuron silencing between 24-72 haH. Late 2nd instar larvae (h after hatching?) were used for expression analysis, while the data indicate that growth retardation upon Gr28a-Gal4 silencing is starting much earlier, possibly even before 24h after hatching. As Gal4 expression patterns can change over time, this suggests that either there is expression of Gr28a-Gal4 in other tissues/neurons that is not blocked by the authors' Gal80 approach (transiently or permanently), or that the other Gal4 lines do not express during the developmental window proposed by the authors to be the main driver of the effect. The whole paper critically relies on the used Gr28a-Gal4 line, which raises some doubts about the specific link to the proposed v'td neurons. Addressing this would require very detailed description of the developmental expression of this line in neuronal and non-neuronal tissues and showing why other v'td neuron-expressing lines do not display the same effect.

We agree with the reviewer that Gal4 expression levels can vary during development, as well as between different Gal4 lines even if they are reported to be expressed in the same population of neurons.
While we cannot fully explain why some other driver lines such as *Gr89a-Gal4* and *Gr28b.c-Gal4* do not cause similar developmental phenotype as *Gr28a-Gal4*, this is likely due to differential expression levels and/or patterns of Gal4 between these driver lines. This point is now explained in the revised manuscript on p. 11.
As for the temporal expression change of *Gr28a-Gal4*, we now observed its detailed expression patterns in both 1st and 2nd instars, along with the two additional Gal4 lines used. The results are now shown in Fig. S2. Expression patterns of all these three drivers are highly consistent between the two stages, suggesting that the expression patterns of these drivers are unchanged during early larval stages.
In order to demonstrate that other Gal4 lines expressed in v'td neurons can also cause similar developmental delay phenotype as *Gr28a-Gal4*, we used two additional driver lines as suggested by the reviewer below in point #2. *R73B01-Gal4* is expressed in v'td2 neurons throughout the body wall segment (Fig. S2) (Qian et al., 2018), while *R22C07-Gal4* is selectively expressed in A4¤A6 v'td2 neurons (Fig. S2) (Qian et al., 2018). Silencing of *R73B01-Gal4*-expressing neurons (*R73B01>Kir2.1*), but not that of *R22C07*-expressing neurons (*R22C07>Kir2.1*), caused a significant delay in larval- prepupal transition to a similar extent as *Gr28a>Kir2.1* animals (Fig. S2). Taken together, there results further support the idea that A1-A3 v'td2 neuron activities are required for normal development.

2. Along the same lines as above, specificity of the authors' findings should be shown using additional independent lines expressing in the described neuronal subset, e.g. 22C07-Gal4 or other lines characterized in Qian et al., J Neurosci 2019.

We thank the reviewer for this suggestion. As explained above, we now used two additional drivers that are expressed in overlapping but different populations of v'td neurons. The results support the idea that A1-A3 v'td2 neuron activities are required for normal development.

3. There is an elaborate description of the involvement of Gr`s in nutrient sensing in the introduction and discussion, thus one would expect that the authors had checked for the

© 2022. Published by The Company of Biologists under the terms of the Creative Commons Attribution License (https://creativecommons.org/licenses/by/4.0/).

involvement of Gr28a v'td neurons in sensing nutrients externally or internally. The authors suggest that Gr28a-expressing v'td cells are involved in RNA sensing as suggested for Gr28a in previous work (Mishra et al., PLOS Biol 2018). However, this was not tested and the conclusion that Gr28a-Gal4 labelled v'td neurons are gustatory receptor neurons regulating ILP production is somewhat overstated. V'td neurons have so far been implicated in sensing CO2 and noxious light (Hückesfeld et al. et al. Elife 2021, Imambocus et al., Curr Biol 2021) yet internal sensory functions as proposed here are not shown by the authors. The authors should keep in mind that Gal4 lines based on enhancer fragments as used in this study do not necessarily represent the expression of the respective Gr, including Gr28a. In several places in the manuscript, this distinction is not so clear and the authors discuss a lot about the role of Gr28a without testing its function, which could have been performed e.g with Gr28a-RNAi.

To address this point raised by the reviewer, we generated a null mutant of *Gr28a* by replacing the whole gene with a Gal4-containing cassette (Fig. S3). Homozygous *$Gr28a^{GAL4}$* mutants showed growth rate reduction and pupariation delay similar to *Gr28a*-expressing v'td neuron-silenced larvae, but the phenotype was much milder (Fig. 4, 5, and S3). This suggests that *Gr28a*-expressing v'td neuron activity is not completely suppressed in *Gr28a* mutants. We also discussed these results in Discussion (p. 16, lines 330-350).

4. The title is overstating the findings ("Internal sensory neurons innervate insulin-producing cells…"), as direct connectivity of Gr28a-Gal4-expressing neurons with IPCs has not been convincingly demonstrated. Close proximity of respective axonal and dendritic arbors is not a sufficient criterion for connectivity. The SEZ is densely packed and many of the neurons with projections in this region are not necessarily directly connected. At the minimum, a synaptic GRASP experiment would be necessary to indicate direct connectivity. The authors could have easily performed this, as these tools are commonly available and standard in the field. Testing functional connectivity, even if indirect, would be an even better proof of a causal link between v'td neurons and IPCs, e.g. testing if activation (e.g. optogenetically) of the identified v'td neurons increases IPC activity.

Based on the reviewer's suggestion, we performed GRASP analysis and the results are now shown in Fig 3E-G''. Reconstituted GFP signals were clearly observed in the SEZ between neurons labeled by *Gr28a-Gal4* and *ilp2-LexA*, indicating physical interactions between *Gr28a*-expressing neurons and IPCs. We also modified the title to avoid overstatement.

5. Body weight seems to be already affected at 24 haH by silencing of Gr28b-Gal4 expressing cells (Fig. 5A). However, statistical analysis for Fig. 5A-D was not performed. This makes it difficult to evaluate the data and effects. The authors claim that the effect at 24h hAH is small, yet at the scale of the figure it is impossible to judge that. Body weight might well be similarly reduced as at later stages. Moreover, Fig. 5C and D are confusing and not well explained in the legend. It looks like weight and surface was simply normalized to 1 at 24h, which does not add any information.

Based on the reviewer's comment, we extensively modified Fig. 5A-D: first, we separated the line plots and jitter plots so the results can be interpreted more easily. Second, we used a log scale for y-axis so the growth rate can be readily appreciated in the line plots. Lastly, we also performed statistical analyses, and the results are shown in Fig. 5B and D.

6. It is not clear to me how Ilp2 levels were quantified. Was there any normalization performed to correct for overall sample differences? The methods lack sufficient detail to reconcile how this was performed.

We thank the reviewer for pointing this out. We now have "Image Analyses" section in Materials and Methods (p. 21), and the quantification method is explained in detail there.

7. No causal link between Ilp2 accumulation in IPCs and the observed transient developmental delay upon v'td neuron silencing has been provided. Can the developmental delay be rescued by increasing IPC activity or Ilp2 release?

As insulin signaling is a central and very strong regulator of insect development, increasing IPC activity or ILP expression always leads to overgrowth phenotype, even if the normal insulin

© 2022. Published by The Company of Biologists under the terms of the Creative Commons Attribution License (https://creativecommons.org/licenses/by/4.0/).

signaling is not disrupted at all. It is therefore not ideal to forcefully activate insulin signaling to evaluate causal relationship. Having said that, we agree with the reviewer that we have not clearly addressed the causality between ILP2 accumulation and developmental delay. We carefully revised the manuscript and avoided any overstatement, including the title of the manuscript.

Minor points

1. Fig 2K'and K'': Gr28a-Gal4 is expressed in v'td and HCG/PVG neurons projecting to the SEZ from the posterior and anterior end, respectively. The axonal bundle from HCG/PVG neurons in the SEZ in Fig. 2K' appears thicker than shown later in Fig. 3C. Are axons from additional (gustatory) Gr28a expressing neurons present as well?

Axon terminals of *Gr28a*-expressing neurons in the pharyngeal (VPS and PPS) and external (TOG and DOG) gustatory organs are visible in lateral areas of the SEZ. We now labeled these axon terminals in Fig. 2K' and explained it in the figure legend.

2. Statistical analysis is only provided for Fig. 5F and G, but not Fig. 5A to D. Moreover, while a supplementary table is appropriate to show all P values, it is very inconvenient to not indicate the relevant P values in the figure. This makes an assessment of the data unnecessarily complicated.

We performed additional statistical analyses for Fig. 5A-D, as well as for other data as suggested by other reviewers. These results are now shown in each figure, along with Table S4 that contains all the raw data from statistical analyses.

We thank the reviewer for their critical but constructive comments on our manuscript.

We would like to thank you and the three anonymous reviewers one last time for all the valuable inputs to our manuscript. We believe that our manuscript has been significantly improved with multiple additional experiments and thorough edits to the text. We sincerely hope that it can now satisfy the broad readership of *Development*.

<u>Second decision letter</u>

MS ID#: DEVELOP/2021/200440

MS TITLE: Internal sensory neurons regulate stage-specific growth in Drosophila

AUTHORS: Yuya Ohhara and Naoki Yamanaka

I have now received all the referees' reports on the above manuscript, and have reached a decision. The referees' comments are appended below, or you can access them online: please go to BenchPress and click on the 'Manuscripts with Decisions' queue in the Author Area.

Both the referees and myself remain enthusiastic about this study, and acknowledge that this revised version is much improved. I do, however, have an outstanding significant concern regarding this point raised by reviewer 3:

"The additional analysis of other v'td neuron Gal4 lines strengthens the authors' notion that A1-A3 v'td2 neurons participate in the observed developmental delay. However, R73B01-Gal4 expresses in a number of gustatory neurons and in the CNS somewhat limiting the specificity of the phenotype in this line. In contrast, Gr28b.c-Gal4 and particularly Gr89a-Gal4 are quite specific for v'td2 neurons, but do not show any defects upon Kir2.1-mediated silencing. The authors (and I) cannot really explain these discrepancies, but in my view, this somewhat limits the specificity of the observed phenotype."

© 2022. Published by The Company of Biologists under the terms of the Creative Commons Attribution License (https://creativecommons.org/licenses/by/4.0/).

Whilst it is laudable that you have used additional lines that target these neurons to resolve the initial discrepancy, the fact remains that two other lines that are seemingly strongly expressed in these neurons do not result in growth delay when inactivated. Consequently, and given that the Gr28 mutants are not developmentally delayed, we cannot be 100% sure that these neurons are "the" relevant neurons.

It seems to be that this puzzling observation needs to be somehow resolved before I can accept this manuscript for publication, given that the manuscript's main message is the finding of a role for these neurons in regulating growth. If the issue is different expression levels of different Gal4 lines as suggested, might using two copies of the Gal4 driver or the UAS-reporter help resolve this? But I would be happy to consider other approaches that aim to answer this question.

If you are able to revise the manuscript along the lines suggested, I will be happy receive a revised version of the manuscript. If it would be helpful, you are welcome to contact me to discuss your revision in greater detail.

Please attend to all of the reviewers' comments and ensure that you clearly highlight all changes made in the revised manuscript. Please avoid using 'Tracked changes' in Word files as these are lost in PDF conversion. I should be grateful if you would also provide a point-by-point response detailing how you have dealt with the points raised by the reviewers in the 'Response to Reviewers' box. If you do not agree with any of their criticisms or suggestions please explain clearly why this is so.

Reviewer 1

Advance summary and potential significance to field

This study investigates the role of chemosensory inputs on growth and development. To identify gustatory receptor neurons that couple the chemosensory system with larval development, the authors employed 66 different Gr-Gal4 lines, whose regulatory elements are derived from corresponding Gr genes, to silence neuronal activity and screened for altered timing of pupariation. Their screen identify Gr28a-Gal4 positive neurons as required for normal larval growth and developmental timing. They go on to characterize these Gr28a-expressing neurons and identify different neuronal populations. Using Gal80 to restrict expression to different subpopulations of neurons and show that the neurons controlling developmental timing correspond to peripheral body wall sensory neurons that project onto the insulin-producing cells. They next show that silencing of the Gr28a-expressing neurons blocks Dilp release from the IPCs and suppress larval growth specifically during mid-larval period.

Numerous studies have shown a link between internal nutrient-sensing mechanisms and Dilp-mediated larval growth. This study provides an interesting novel link between the chemosensory system and larval growth.

Comments for the author

This reviewer is satisfied with the experiments carried out to address the points raised in during the initial revision.

Reviewer 2

Advance summary and potential significance to field

Overall, this is a very nice study that provides new insights into the neuronal control of insulin signaling and growth. I think it will be of interest to those interested in growth control and in Drosophila physiology and development.

Comments for the author

The authors have addressed all my comments in this revised version. I'm happy to recommend the paper for publication. Congratulations to the authors on a very nice story.

© 2022. Published by The Company of Biologists under the terms of the Creative Commons Attribution License (https://creativecommons.org/licenses/by/4.0/).

Reviewer 3

Advance summary and potential significance to field

Using a screening approach, the authors show that Gr28a-Gal4 expressing internal sensory neurons seem to be transiently involved in developmental progression of Drosophila larvae. Gr28a expressing neurons (v'td and HCG/PVG) project near IPC dendrites in the SEZ as shown by GRASP experiments. The authors confirm that Gr28a plays some role in this phenotype, even though knocking out Gr28a gives rise to a weaker developmental delay. The authors provide data that insulin release (Ilp2 protein and InR mRNA levels in IPCs and p-Akt signal in peripheral tissues) is reduced upon silencing of Gr28a-Gal4-positive cells. Feeding is not affected in these animals suggesting food intake-independent delay of development. The authors propose that internal sensory neurons via expressed gustatory receptors transiently regulate Insulin release to promote developmental progression of Drosophila larvae.

Comments for the author

The authors made a valiant effort in addressing the concerns and I would like to thank them for taking the (constructive) criticism seriously. While I still have some doubts about the meaning and the specificity of the observed phenotypes, I think the manuscript is much improved and the authors extensively addressed most of the points raised. I would like to spell out some limitations of the study for further consideration, but not for mandatory revisions, as the authors revised the wording of their manuscript carefully and adequately. If a section of “Limitations of this study” can be included some of the points might be mentioned though.

1. While the addition of a GRASP experiment to show direct proximity of Gr28a expressing neurons to IPCs Is helpful, there are better tools available to show synaptic connectivity. Cell surface marker-mediated GRASP is prone to adhesion artefacts, limiting the interpretation of direct synaptic contact. An activity-dependent Syb-GRASP experiment (MacPherson et al Nat Comm 2015) would have been a better and more specific approach for showing direct synaptic connectivity.

2. The additional analysis of other v'td neuron Gal4 lines strengthens the authors' notion that A1-A3 v'td2 neurons participate in the observed developmental delay. However, R73B01-Gal4 expresses in a number of gustatory neurons and in the CNS somewhat limiting the specificity of the phenotype in this line. In contrast, Gr28b.c-Gal4 and particularly Gr89a-Gal4 are quite specific for v'td2 neurons, but do not show any defects upon Kir2.1-mediated silencing. The authors (and I) cannot really explain these discrepancies, but in my view, this somewhat limits the specificity of the observed phenotype.

3. Gr28a neurons likely coexpress several other Grs (e.g. Gr28b.c, Gr89a) so it might not be surprising that the Gr28a knockout larvae do not show the same developmental delay as their silencing.

4. The more detailed description of the analysis of Ilp2 and p-Akt levels is helpful. However, I would have preferred to have an internal staining control that can be used for normalization. Immunohistochemical quantification is tricky as staining intensities vary between animals and depend on the preparation, size of the animal, mounting etc. Maintaining the same microscope setting is in my view not sufficient. p-Akt levels are typically normalized to total Akt (at least in Western Blots), and Ilp2 signal could have been normalized either to GFP marker expression or a general co-stained protein (e.g. actin or similar).

© 2022. Published by The Company of Biologists under the terms of the Creative Commons Attribution License (https://creativecommons.org/licenses/by/4.0/).

Second revision

Author response to reviewers' comments

Reviewer 3:

"The additional analysis of other v'td neuron Gal4 lines strengthens the authors' notion that A1-A3 v'td2 neurons participate in the observed developmental delay. However, R73B01-Gal4 expresses in a number of gustatory neurons and in the CNS somewhat limiting the specificity of the phenotype in this line. In contrast, Gr28b.c-Gal4 and particularly Gr89a-Gal4 are quite specific for v'td2 neurons, but do not show any defects upon Kir2.1-mediated silencing. The authors (and I) cannot really explain these discrepancies, but in my view, this somewhat limits the specificity of the observed phenotype."

And here is your suggestion in response to the reviewer's comment above:

Whilst it is laudable that you have used additional lines that target these neurons to resolve the initial discrepancy, the fact remains that two other lines that are seemingly strongly expressed in these neurons do not result in growth delay when inactivated. Consequently, and given that the Gr28 mutants are not developmentally delayed, we cannot be 100% sure that these neurons are "the" relevant neurons.
It seems to be that this puzzling observation needs to be somehow resolved before I can accept this manuscript for publication, given that the manuscript's main message is the finding of a role for these neurons in regulating growth. If the issue is different expression levels of different Gal4 lines as suggested, might using two copies of the Gal4 driver or the UAS-reporter help resolve this? But I would be happy to consider other approaches that aim to answer this question.

We checked the expression patterns of *Gr28b.c-Gal4* and *Gr89a-Gal4* in both first and second instars, and the results are now shown in Figure S2. This confirmed their expression in v'td neurons, although *Gr28b.c-Gal4* is also expressed in many other peripheral neurons. In the same figure, we also presented the results of neuronal silencing experiments, where the copy number of *Gal4* and/or *UAS- Kir2.1* was doubled to enhance the silencing effects (Fig. S2K). The results are explained on p.11.

Just to correct a small misconception by the reviewer (which is our fault as we did not emphasize it in the original manuscript), neuronal silencing using *Gr28b.c-Gal4* indeed causes a significant developmental delay (Fig. 1 and S2K), and this was slightly augmented when two copies of *Gr28b.c- Gal4* and *UAS-Kir2.1* were introduced (Fig. S2K). Similarly, although *Gr89a-Gal4*-mediated silencing did not cause a significant developmental delay when a single copy of *UAS-Kir2.1* was used, introducing a second copy of *UAS-Kir2.1* caused a significant delay in pupariation (homozygous *Gr89a- Gal4* animals are lethal). These results thus further support our model that v'td neurons are responsible for controlling larval growth in flies.

We do admit though that the strength of the delay phenotype is not perfectly comparable among different drivers, even when the copy numbers of the transgenes were doubled for some of them. As we discussed in the text, we attribute this difference to differential expression levels and patterns of the drivers. This is particularly true for *Gr28b.c-Gal4*, which is expressed in many different types of peripheral neurons as we showed in Figure S2 - we have no idea how silencing of these additional neurons affects developmental timing. As you know, such variation of phenotypic severity is common when using multiple Gal4 drivers with overlapping expression patterns, and standardizing their expression levels in a particular population of cells while eliminating side effects from others is often impossible. At the minimum, we have successfully shown that neuronal silencing using all the known drivers expressed in A1-A3 v'td neurons (*Gr28a-Gal4*, *Gr28b.c-Gal4*, *Gr89a-Gal4*, and *R73B01-Gal4*) leads to developmental delay with varying degrees, and we can explain potential reasons of the phenotypic variability. We hope that this can dispel the remaining concern about our manuscript.

We would like to thank you and the anonymous reviewers one last time, particularly Reviewer 3 in this second round of revision, for all the valuable inputs to our manuscript. We hope readers of our paper would appreciate our multifaceted approach, such as limiting the expression of

© 2022. Published by The Company of Biologists under the terms of the Creative Commons Attribution License (https://creativecommons.org/licenses/by/4.0/).

Gr28a-Gal4 with multiple *Gal80* lines, confirming the connection of *Gr28a*-positive v'td neurons with the IPCs, and demonstrating the ILP release and larval growth phenotypes caused by *Gr28a*-positive v'td neuron silencing. We strongly believe that our revised manuscript can satisfy the broad readership of *Development*, and we sincerely hope that you feel the same.

Third decision letter

MS ID#: DEVELOP/2021/200440

MS TITLE: Internal sensory neurons regulate stage-specific growth in Drosophila

AUTHORS: Yuya Ohhara and Naoki Yamanaka
ARTICLE TYPE: Research Article

I am happy to tell you that your manuscript has been accepted for publication in Development, pending our standard ethics checks. I have appended one of the reviewers' comments below - you may want to consider including some of their thoughts/comments in your manuscript but I will leave it to you to decide if/how you would like to do this; acceptance is not conditional on this but it might be useful for the future readers of your paper. I would like to thank you and co-authors for substantially improving the manuscript through the review process. Congrats on a nice story!

Reviewer 3

Advance summary and potential significance to field

As stated before, this is an interesting study on the developmental requirement of specific sensory neurons to promote larval development in Drosophila.

Comments for the author

The authors did thorough additional experiments that addressed my main concern with their conclusion, which is now much more convincing. While some limitations remain (phenotype variability depending on the line and none is as strong as the Gr28a-Gal4 effect), and while Gr28a itself might not be the only Gr involved in the described phenomenon, they do show an interesting developmental aspect mediated by these neurons.
Just as a note: Gr28b.c-Gal4 is not expressed in that many more cells compared to the other lines used in the assays. It has been reported to be expressed in class IV da and v`td2 neurons, with some expression in few cells in the VNC. The authors also describe expression in the foregut and based on the images the body wall/CNS expression pattern is confirmed. Class IV da neuron silencing has no known effect on developmental timing, although it might not have been investigated in detail.

© 2022. Published by The Company of Biologists under the terms of the Creative Commons Attribution License (https://creativecommons.org/licenses/by/4.0/).
